# Inside Out: A Scoping Review on the Physical Education Teacher’s Personality

**DOI:** 10.3389/fpsyg.2019.02510

**Published:** 2019-11-08

**Authors:** Melina Schnitzius, Alina Kirch, Filip Mess, Sarah Spengler

**Affiliations:** Department of Sport and Health Sciences, Technical University of Munich, Munich, Germany

**Keywords:** personality, teacher, coach, physical education, school, sports, scoping review, teaching competence

## Abstract

The teacher’s personality in general plays an important role in the educational process. It is often examined in relation to outcome factors on the teacher or student side, e.g., teaching effectiveness or student motivation. Physical education (PE) with its peculiarities and allocated educational mandate particularly demands the personality of the PE teacher. Research considering this group of teachers is sparse, diverse and hard to capture due to different personality understandings. Our review therefore aims at identifying and analyzing underlying personality understandings, research questions and results of studies considering the personality of the PE teacher. We conducted a scoping review. After the screening and additional analyses process, 23 studies were included. Included references had to be empirical, published in German or English and explicitly examine the PE teacher’s personality as variable or mention it as outcome factor in school context. All studies are cross-sectional, 22 studies quantitative, one qualitative. Regarding personality understandings, 12 studies follow a trait psychological, six studies a vocational, one study an interpersonal personality understanding. Four studies’ personality understanding is not concretely determinable. Considering research questions, three studies aim at identifying the PE teacher’s personality in general and do, e.g., not find considerable differences between the PE teacher’s and other teacher’s personality. Nine studies examine the relationship between the PE teacher’s personality and different correlates such as burnout, highlighting, e.g., that female PE teachers’ burnout process is less homogeneous than males. Eleven studies examine the PE teacher’s personality from an external view and show, e.g., that students of different age groups perceive the PE teacher’s personality differently. Our review offers possible practical implications. By e.g., knowing their personality structure – their *inside* –, PE teachers can play to their own strengths and make use of their individual personality configuration in order to teach authentically and successfully, i.e., transferring the *inside* to the *outside*. Due to partly questionable and fragmentary methodologies of the included studies, results have to be interpreted with caution. More studies considering the PE teacher’s personality following a broad personality understanding are needed to include potentially relevant factors for teaching and by this receive evident insights.

## Introduction

The teacher – one key player in the educational process in school – naturally attracts attention in didactic approaches. The teacher’s role – e.g., as one axis in the well-recognized didactic triangle – and by this his general impact within the students’ learning process is undisputed. General models of education such as Helmke’s (2017) *Utilization of learning opportunities model*, which depict power factors of good lessons, also highlight the teacher’s role and among this the teacher’s personality and its influence on the quality of lessons. Traditional models of professional teaching competence also include the teacher’s personality and make it a priority among other essential factors. Dunkin and Biddle’s (1974) internationally well-recognized *Conceptual model of factors influencing teaching and learning*, e.g., attributes the teacher’s properties (skills, intelligence, motivations and personality traits) a substantial role among variables predicting lesson and learning success. Considering German educational research, in Baumert and Kunter’s (2013) *Model of professional teacher competence* four facets constitute the teacher’s ability to perform: motivational orientations, self-regulation, beliefs/values/goals and professional knowledge. Professional teaching practice is seen as result of the coaction of these facets (Baumert and Kunter, 2013). Except for the latter one, personality characteristics play an important role in these facets. Baumert and Kunter’s (2013) model allows for the development of professional competence over time, but explicitly highlights the role of relatively stable, implicit factors such as personality characteristics within the professional development process. Personality characteristics influence firstly the uptake of learning opportunities, thereby the teacher’s professional competence and finally their professional practice (Kunter et al., 2013a). The teacher’s individual personality characteristics therefore are essential for succeeding in teacher education and the teaching career.

Research on the relationship between the teacher’s personality and their performance has a particularly long tradition. Succeeding as a teacher encompasses and is often measured by teacher-related factors such as academic success, satisfaction in teaching, teacher well-being or student-related factors such as student motivation or student achievement. General educational research often examines explicitly the relationship between the teacher’s personality and the aforementioned *success factors*: On the teacher side, e.g., satisfaction in teaching, teacher burnout, teacher self-efficacy or teacher effectiveness (Mayr, 2011; Djigić et al., 2014; Cramer and Binder, 2015; Perera et al., 2018; Kell, 2019; Kim et al., 2019). On the student side, teacher personality is often analyzed in relation to student motivation or student achievement (Wayne and Youngs, 2003; Hattie, 2009; Jahangiri, 2016; Khalilzadeh and Khodi, 2018; Kim et al., 2018, 2019). Kim et al. (2018) attribute the identification of vital factors of the teacher’s personality a promising role for their effectiveness – measured by teaching performance. Knowing about vital personality factors can be beneficial for teaching in general, e.g., for teacher’s planning and reflection of lessons – as indicated in the teacher’s role in models of lesson planning and evaluation (Döhring and Gissel, 2016). It might also be helpful for the initial teacher selection or hiring process (Bastian et al., 2017; Kell, 2019).

In order to first understand the role and impact of the teacher’s personality for the educational process, the term personality has to be defined and appropriate understandings have to be considered. Such a clear understanding serves as a basis for deriving possible practical implications for teaching or even structural and organizational implications. Following Pervin and Cervone (2008) the term personality refers to “psychological qualities that contribute to an individual’s enduring and distinctive patterns of feeling, thinking and behaving.” In order to understand the construct of personality and ensure its comprehensibility, researchers have created models or frameworks. Even though personality psychology still lacks a comprehensive and universal framework for understanding the whole person, Costa and McCrae’s *Five Factor Model (FFM)* (Costa and McCrae, 1999) has gained excessive attention (McAdams and Pals, 2006). This prevailing and widely accepted model follows a multidimensional understanding, clustering personality characteristics in the five facets: *Openness*, *Conscientiousness*, *Extraversion*, *Agreeableness* and *Neuroticism (OCEAN)* (John et al., 2008). These factors define a person’s personality on a very global level (Rammstedt et al., 2018). The *FFM* is often used interchangeably with the term *Big Five*. The two frameworks are very similar but can be differentiated from each other regarding their origin: the *FFM* has been developed by empirically analyzing personality questionnaires whereas the *Big Five* are based on a lexical approach (Kim et al., 2019) believing that distinguishing characteristics have their origin in natural language use (Goldberg, 1981). Both frameworks share the understanding of personality by the use of five independent and bipolar categories (Rammstedt et al., 2018) and currently dominate personality research. Next to the aforementioned classical trait psychological personality understanding, personality research also borders upon other approaches such as the interactionist understanding. Here personality together with the situation determines an individual’s behavior (Swann and Bosson, 2010). This understanding of personality can be considered less static. Moreover, considering personality research focusing on a specific professional group, Holland’s (1997) theory and model of vocational personality can be seen as outlasting and prevalent in the occupational context. Holland characterizes people regarding their fit to six different personality types (*Realistic, Investigative, Artistic, Social, Enterprising, Conventional – RIASEC*) and highlights the influence of the environment and by this – similar to the interactionist understanding – developmental possibilities of the worker’s personality. Even though originating from different understandings, all exemplary illustrated approaches claim to assess personality. In addition to following traditional and established approaches, further personal facets such as care and enthusiasm are often considered as closely associated or even equated with personality.

Examining the teacher’s personality is common practice in general educational research. Göncz (2017) conducted a scoping review and aimed at giving an overview of research activities concerning the teacher’s personality and by this highlighting strategies for educational psychology. Göncz (2017) identified five types of studies classified according to their research questions: Studies of teacher typologies; Studies of teachers’ desirable and undesirable features; Studies of teachers’ professional behaviors and their influence on students; Studies of teachers’ professional identity and Studies of teacher personality within the framework of personality theories (particularly within the *FFM*). In the conclusions Göncz (2017) takes position regarding the merits of the identified groups and proclaims the findings from studies following traditional personality theories “as the best starting point for a more comprehensive psychological theory of teacher personality in educational psychology.”

Considering the personality of the physical education teacher (PET), Miethling and Gieß-Stüber (2007) also stated the PET’s personality as pivotal point of their professional competence. This becomes especially important in conjunction with physical education (PE)’s allocated educational mandate. PE’s mandate postulates (a) to educate the students’ physical – e.g., by developing physical fitness and ideally a lifelong engagement in sports and (b) to educate through the physical – e.g., developing students’ personality, fostering value imparting and moral education (Sallis and McKenzie, 1991). It is essential that PETs initially reflect their individual prerequisites and potentials (e.g., strengths and weaknesses, personality characteristics) in order to better understand and approach their students. PETs on the one hand have to reflect their own understanding of sports and teach this understanding their students to engage them in sports. On the other hand, PETs have to reflect their own values and then impart these values on their students to educate them beyond the physical. If they manage to fulfill both tasks, they are most likely able to successfully implement PE’s aforementioned dual mandate. PETs serve as role models physically and by conveying their own reflected mission statement to their students. How PETs are perceived by their students in this process certainly depends on their personality. Beyond the challenging educational mandate, PETs are faced with further challenges that demand their personalities. The proximity between the PET and their students poses a challenge that requires the PET’s personal characteristics. PETs need to address each child’s needs, challenge each child at their personal level and create a positive, secure and supportive relationship in a climate where learning can succeed. This is among others achieved by PETs who know their personal qualities, reflect them and convert this process into empathetic, enthusiastic and ideally sustainable teaching. Considering the PET’s personality – the *inside* – should therefore receive special attention among personality research in school context. Knowing the teacher’s *inside* and transferring this to the *outside* – making it visible – can then support lesson planning and teaching.

Similar to research concerning teachers in general, in studies on the PET’s personality the term personality though is construed differently and analyzed in various contexts with different correlates. Contrary to general educational research, a review article summarizing international publications concerning the PET’s personality is missing. A review article is necessary though in order to organize the prevailing picture of the understanding of the PET’s personality – its definition, characteristics or related factors –, its correlates and by this its possible impact on educational outcomes. Therefore this review aims at answering the following research question: What are the underlying personality understandings, research questions and results of studies considering the personality of the PET in school?

## Methods

In order to answer the above stated research question, we conducted a scoping review. In 2005 Arksey and O’Malley outlined a first framework for this review approach. Arksey and O’Malley (2005) follow Mays et al.’s (2001) definition – assigning scoping studies the opportunity and task to easily depict a research area’s fundamental specifics. They generally attribute scoping studies a comprehensive coverage. Our decision to conduct a scoping review was based on three reasons: First, as preliminary literature searches on the PET’s personality revealed that research in this field is diverse and the understanding of personality vague, a scoping review that typically does not try to find an answer to a specific question but summarizes what questions have been asked, seemed to be appropriate (McEvoy et al., 2015; García-Moya et al., 2018). Second, we were interested in the identification of certain characteristics or concepts related to personality and in mapping, reporting or discussing these with finally suggesting practical implications – according to Munn et al. (2018) indications for a scoping review and therefore again supporting our decision. Third, conducting scoping reviews has become more popular in the educational context with a couple of recent perceptive scoping reviews published (e.g., McEvoy et al., 2015; Göncz, 2017; Richards et al., 2017; Sperka and Enright, 2017; Robinson, 2018; Killian et al., 2019).

Our research team consisted of two researchers. We independently passed through the individual phases of the review process following Arksey and O’Malley’s (2005) six stages of their methodological framework: (1) Identifying the research question; (2) Identifying relevant studies; (3) Study selection; (4) Charting the data; (5) Collating, summarizing and reporting results; and (6) Consultation. Conflicts were cleared collaboratively after each step.

### Stage 1: Identifying the Research Question

Considering Arksey and O’Malley’s (2005) possible purposes of a scoping review, our review followed mostly two purposes: *Examine the extent, nature and range of research activity* and *identify research gaps in the existing research*. Due to the fact that preceding research on the PET’s personality revealed inconsistency concerning the understanding and interpretation of personality, we decided to keep our research question relatively wide. We focused on ascertaining what type of empirical literature exists dealing with the personality of the PET in school, which understandings of personality are pursued and which questions are asked considering the personality of the PET in school. In order to capture most interpretations of the ambiguous term personality we did not specify it and decided to follow an open personality understanding. This allowed for different understandings to be included in our review and by this receive an unaffected and true picture of the existing literature. We deliberately aimed at summarizing literature that either claims to assess personality as a variable or mentions personality as an outcome. Thus, the review’s inclusion criteria were the following: content = personality, setting = PE, participants = PETs (personally or via external view), publication language = English or German.

### Stage 2: Identifying Relevant Studies

In order to answer the research question we developed the search string, including three main categories: (1) Content: *Personality*; (2) Participants: *PETs*; (3) Setting: *PE.* Aiming at English and German publications, we included both languages in our search string:


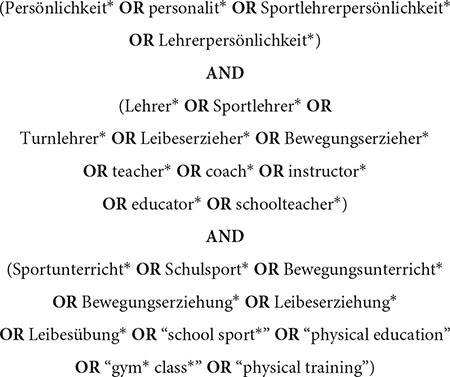


Category 1 (Content) was searched on title/abstract level as personality had to be an essential part in the potentially included text. Category 2 (Participants) and category 3 (Setting) were searched on full-text level. Initially, no restriction regarding the publication date was undertaken. We chose a comprehensive selection of eleven approved databases in the field of school sport research covering English and German texts: Education Source, ERIC, PsychARTICLES, PsycINFO, PSYNDEX, PubMed, Scopus, SocINDEX, SPOLIT, SPORTDiscus, Web of Science. The initial database search was undertaken on February 6th 2017. On June 12th 2018 we fulfilled update search one and on April 11th 2019 update search two.

### Stage 3: Study Selection

**FIGURE 1:**
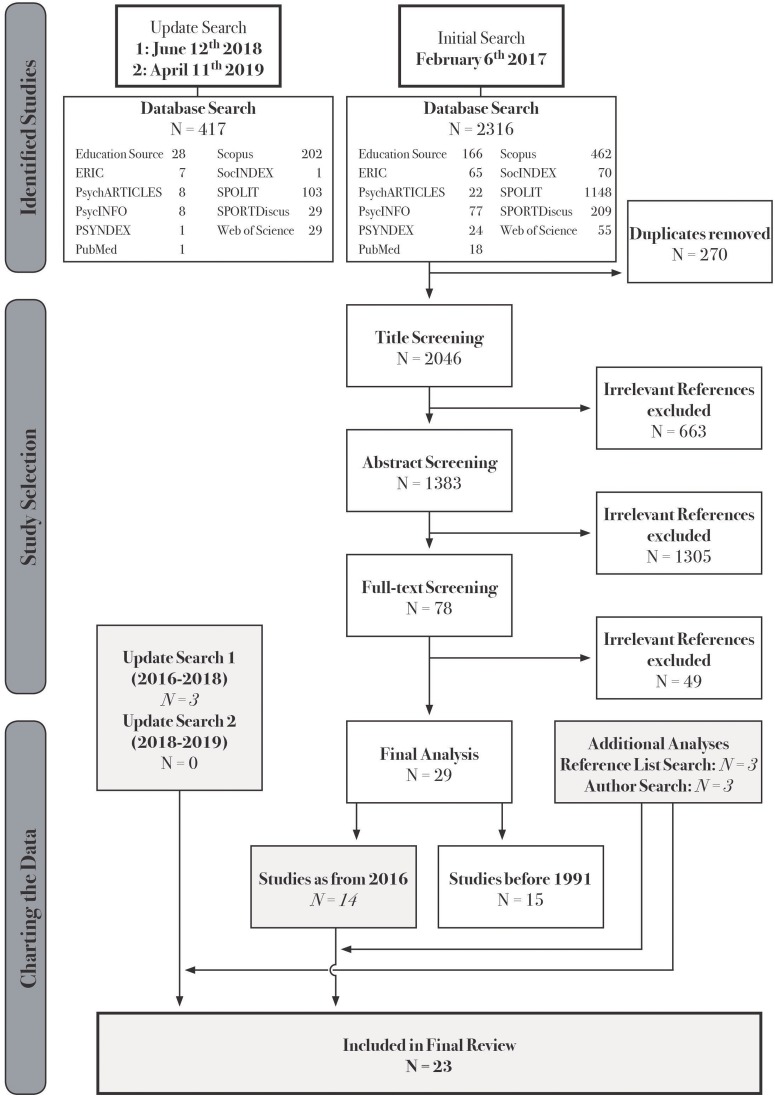
Flow chart of the search and reference selection process.

After removing duplicates, we independently screened the titles. References were excluded if they clearly did not examine PETs (personally or via external view), if the setting clearly was not PE or if the content clearly was not personality. After screening titles, the remaining abstracts were screened. First, we deployed the same exclusion criteria as before. Screening abstracts allowed identifying non-empirical studies, which were excluded. As we aimed at providing a broad picture of the existing literature, we kept our search strategy rather wide and our exclusion criteria quite soft. If references belonged to editorial works, these were provided and screened for chapters containing empirical studies. Finally, the full-texts of the remaining studies were provided and independently screened applying the same exclusion criteria as before (excluded if: not in English or German, not empirical, not examining PETs, not school setting, not personality). Ultimately, we searched the reference lists of all finally included texts and examined other work of the authors. We screened the authors’ websites and publication lists for additional relevant texts and checked for conference presentations and projects. In this process, the same exclusion/inclusion criteria as in the initial search were applied. We created a flow chart which documents the search and reference selection process (see Figure 1).

### Stage 4: Charting the Data

We independently extracted relevant data from the included texts and collaboratively agreed on a presentation format representing the studies’ key information. This step was conducted according to the methodological guideline of the Joanna Briggs Institute (Peters et al., 2015). A table was created which served as the basis for comparing and contrasting the included texts (see Table 1).

**TABLE 1:** Included studies as from 2006.

**Author (year) origin**	**Study design/method sample**	**Aim**	**Personality inventory**	**Personality understanding (representative)**	**Main results**
***(I) The PET’s personality (N* = *3)***					
García-Villanueva et al. (2017) Mexico	^∗^cross-sec./quant. ^∗^53 PETs (35 m.)	To analyze diff. among PETs in the 4 gender-related pers. scales of *IMAFE* and work variables sex, age and marital status	quest.: *IMAFE* (Lara Cantú, 1993) ^∗^4 factors: *masculinity, femininity, machismo, submission* = 60 items associated with pers.	Not determinable → gender-related (pers. part of gender-related characteristics)	^∗^no diff. in the 4 scales proposed by IMAFE in regard to pers. char. and work variables sex, age and marital status in the group of PETs
Hassan et al. (2016) India	^∗^cross-sec./quant. ^∗^20 m. PETs, 20 m. OSTs	To measure and compare the *Big 5* pers. factors among m. PETs and OSTs	quest.: *Big 5* Pers. Inventory (Buchanan et al., 2005) ^∗^5 factors *(O, C, E, A, N)* = 20 items	Trait psychological – *Big 5*	^∗^no diff. betw. the *Big 5* pers. factors among PETs and OSTs (valid for all 5 factors)
Mantu and Montu (2014) India	^∗^cross-sec./quant. ^∗^50 m. PETs, 50 m. OSTs	To compare the pers. traits of m. PETs and OSTs	quest.: Eysenck Pers. Quest. (*EPQ-R S*) (Eysenck et al., 1985) ^∗^4 factors: *N, E, psychoticism, lying* = 48 items	Trait psychological (Eysenck)	^∗^no diff. in the pers. factors (means of all factors) betw. PETs and OSTs ^∗^diff. in subc. *E* betw. PETs & OSTs PETs more extraverted
***(II) The PET’s personality and correlates (N* = *9)***					
Arbabisarjou et al. (2016) Iran	^∗^cross-sec./quant. ^∗^60 PETs – from boys high schools	To assess the relations. betw. PET’s pers. and stud.’ individual and social beliefs and activities	quest.: *NEO-FFI* (McCrae and Costa, 2004) ^∗^5 factors: *O, C, E, A, N* = 60 items	Trait psychological – *Big 5* (McCrae and Costa)	^∗^relations. betw. pers. aspects of PETs and stud.’ beliefs and activities ^∗^ corr. for *E* and *O*; no corr. for *N, A, C* ^∗^*E* and *O* together can predict 0.88% of changes of stud.’ beliefs and activities
Brudnik (2007) Poland	^∗^cross-sec./quant. ^∗^160 PETs (77 m.) – prim., sec., post-sec.	To define the vocational pers. profile of PETs and examine diff. reg. gender, work environment and school type (state or priv.)	quest.: *SDS* (Polish version (Lacala et al., 2002) based on Holland (1994) ^∗^activities, skills, occupations; double self-evaluation I and II → 6 scales each = 288 items	Vocational (Holland)	^∗^vocational pers. code differs betw. f. *(Social Investigative Artistic* = *SIA) and* m. *(Social Realistic Enterprising* = *SRE)* PETs ^∗^neither work environment nor type of school influences the obtained results
Brudnik (2010) Poland	^∗^cross-sec./quant. ^∗^333 OSTs (65 m.) + 62 PETs (29 m.) – 22 sec. schools	To ascertain to what degree work-related stress, self-efficacy, prof. pers. determine burnout in OSTs and verify a hypothesis that PETs burn out in keeping with a prof. specific macro-path	quest.: *SDS* (Polish version (Lacala et al., 2002) – based on Holland (1994) ^∗^see Brudnik (2007)	Vocational (Holland)	^∗^m. PETs exhibit typical burn out process for prof. group ^∗^f. PETs burn out less homogenously → macro-paths of m. and f. PETs verified ^∗^burnout process OSTs diff. compared to PETs → disciplinary problems as causal, self-efficacy as preventive factor of burnout among OSTs ^∗^vocational pers. only slightly impacts burnout process
Demir (2014) Turkey	^∗^cross-sec./quant. ^∗^296 PETs (187 m.) – state and priv. sec. schools	To evaluate the relations. betw. pers. traits of PETs in relation to their sports branches and sports types and investigate diff. reg. gender, school type and years of service	quest.: *PERI* (short form of Sevinç, 2005) ^∗^5 factors: *O, responsibility, E, compatibility, emotional stability* = 25 items	Trait psychological	^∗^no diff. betw. PETs’ pers. traits and sports branches, sports types, gender, school type and years of service
Demir (2015a) Turkey	^∗^cross-sec./quant. ^∗^92 volunteer PETs (59 m.) – state and priv. sec. or high schools	To examine the relations. betw. PPC of PETs and gender, school type, school level, years of service and sports branches	quest.: *PET PPC scale* (adapted to PETs by Demir (2012) from Büyüknacar (2008) ^∗^4 subc.: *prof. enthusiasm/dedication; respect for human dignity/justice; stimulating interaction; reflective interaction* = 60 items	Vocational	^∗^PETs see their PPC “completely adequate” → mean scores of *respect for human dignity/justice* subc. lower than other subcomponents ^∗^gender, years of service, sports branches: no diff. ^∗^school type: diff. → priv. school PETs score higher on PPC ^∗^school level: diff. in *stimulating interactions and reflective interactions* (high school PETs score higher on PPC)
Demir (2015b) Turkey	^∗^cross-sec./quant. ^∗^92 PETs (59 m.) – state and priv. sec. and high schools	To evaluate the relations. betw. pers. traits of PETs and their sports branches, sports types	quest.: *PERI* (short form of Sevinç, 2005) ^∗^see Demir (2014)	Trait psychological	^∗^sports branches: corr. only in terms of *emotional stability and compatibility domains* → e.g., racket sports and handball players *emotionally more stable* than gymnasts → e.g., basketball and defense sport players more *compatible* than swimmers ^∗^sports types: no diff.
Hosein Razavi et al. (2012) Iran	^∗^cross-sec./quant. ^∗^162 PETs	To examine if entrepreneurial organizational culture is related to PETs’ entrepreneurial pers. char.	No information	Vocational	^∗^neg. corr. betw. *creative innovation, cooperation, tolerance of creative talents* (parts of entrepreneurial organizational culture) and PETs’ entrepreneurial pers. char. ^∗^pos. corr. betw. *organizational risk-taking, open communication* and PETs’ entrepreneurial pers. char.
Makhmutova et al. (2017) Russia	^∗^cross-sec./quant. ^∗^64 PETs – general educational schools (37 high (= qualified) and 27 low-ranking)	To explore the specifics of mental burnouts in the context of pers. development of the PETs versus their prof. competency levels	quest.: *Cattell’s 16 Pers. Factor* (Form C of 16PF) (Cattell et al., 1993) ^∗^105 items	Trait psychological (Cattell)	^∗^PETs higher on intellectual development less satisfied with work conditions → more likely to burnout ^∗^highly prof. teachers with highest practical experience = most prone to mental burnouts ^∗^qualified PETs exhibit higher rates in the subs. *reasoning* and *emotional stability*
Maryam et al. (2017) Iraq	^∗^cross-sec./quant. ^∗^250 PETs (140 m.)	To examine the relations. betw. (a) burnout and mental health, (b) burnout and pers. traits among PETs	quest.: *NEO-PI-R* (Costa and McCrae, 1992) ^∗^5 factors: *O, C, E, A, N* = 240 items	Trait psychological – *Big 5* (McCrae and Costa)	*mental health, E, O, A* = relevant for burnout process ^∗^neg. corr. betw. burnout subs. and *mental health, E, O* and *A* ^∗^pos. corr. betw. burnout subs. and *N*
***(III) The PET’s personality from an external view (N* = *11)***					
Brandl-Bredenbeck (2006) Germany	^∗^cross-sec./quant. ^∗^8863 stud. – different school levels *– SPRINT*, (Brettschneider, 2006)	To examine stud.’ attitudes toward PETs	quest.: 2 inventories ^∗^semantic differential evaluating PETs = subject- and pers. related: 14 adj. pairs ^∗^PET care = 13 items	Not determinable → generally speaking behavioral + aspect of care	^∗^stud. in general evaluate their PETs pos. → PETs perceived as *self-confident, caring, friendly* ^∗^stud. age diff.: younger stud. evaluate more pos. ^∗^PET age diff.: younger PETs are evaluated better
Demir (2015c) Turkey	^∗^cross-sec./quant. ^∗^1254 stud. - 9th, 10th, 11th grade – 17 schools (16 state, 1 priv.)	To examine how PPC of PETs is perceived by 9th, 10_th_, and 11th grade stud.	quest.: *PET PPCS-Student* [adapted to PETs by Demir (2012) from Büyüknacar (2008)] ^∗^4 subc.: *prof. enthusiasm/dedication; respect for human dignity/justice; stimulating interaction* and *reflective interaction* = 60 items	Vocational	^∗^gender: diff. only in some subc. → *prof. enthusiasm/dedication* and *motivational interaction*: girls more satisfied than boys; *reflective interaction:* vice versa) ^∗^school type: diff. → priv. school more satisfied ^∗^class: diff. betw. 9th, 10th, 11th graders for all subc. → 9th graders evaluate PETs’ PPC most pos.
Lauritsalo et al. (2015) Finland	^∗^cross-sec./quant. ^∗^Finnish stud. communicating in internet forums (356 messages from 9 forums)	To examine what kinds of extrinsic factors underlie opinions expressed in internet discussion forums on experiences of PE: what is the role of the PET, class environment, curriculum and assessment in these opinions?	messages taken from internet discussion forums analyzed by qualitative content analysis	Not determinable generally speaking behavioral	^∗^6 extrinsic factors identified: PET’s pers./behavior = strongest factor containing most statements (40% = 163 messages); 2_nd_ = class environment (24%), 3_rd_ = curriculum (16%), 4_th_ = assessment (9%), facilities & equipment (8%), out-of-school influence and other factors (3%) ^∗^mostly neg. statements and strong feelings of compulsion, humiliation in most opinions (PETs seen as not supportive) 61% of messages in neg. category; 8% pos.; 31% both pos. and neg.
Senn et al. (2017) Austria	^∗^cross-sec./quant. ^∗^122 stud. (87 m.) – year 12, 13 and uni. stud.	To examine how pers. char. and competencies of PETs influence stud. motivation in PE and indicate diff. reg. gender and sportiness	quest.: self-dev. (NN) ^∗^complex 1: imp. of social behavior (PET to stud.) ^∗^complex 2: PET achievement orientation	Not determinable → generally speaking behavioral	^∗^PETs’ social-emotional pers. char. and competencies = imp. for stud. motivation ^∗^gender diff. → PETs realizing stud. fear = more imp. for girls ^∗^partly diff. for sportiness → e.g., *achievement orientation* and *strict grading* more imp. for sporty/active kids
Demir (2016) Turkey	^∗^cross-sec./quant. ^∗^1421 stud. (728 m.) – 6th-8th grade – public and priv. schools	To examine the PPC of PETs as evaluated by stud. and to investigate diff. based on stud.’ gender, school type and class	quest.: *PET PPCS-Student* [adapted to PETs by Demir (2012I from Büyüknacar (2008)] ^∗^see Demir (2015c)	Vocational	^∗^PPC highest average points: “She/he cares that honesty and trust form the basis of our communication at school” = evaluated as “completely adequate” ^∗^PPC lowest average points: “She/he does not criticize a student who exhibits negative behavior in front of the class” = evaluated as “partly adequate” ^∗^gender: diff. only for *motivational interaction* → girls more satisfied than boys ^∗^school type: diff. in all subc. betw. state and priv. school stud. → priv. school stud. in general more satisfied with PPC ^∗^class: diff. in all subc. → 7th graders more satisfied with PETs’ PPC than 6th and 8th graders
Georgiev (2016) Bulgaria	^∗^cross-sec./quant. ^∗^76 stud. (30 m.) – 5th, 6th, 7th grade – sec. school	To reveal stud.’ attitudes toward the prof.-personal qualities and interpersonal char. of the PET pers. and examine if there are diff. reg. the stud.’ age, gender or sports participation	quest.: *Test of T. Leary* (Leary, 2004) and self-dev. quest. (stud.’ attitudes toward PETs’ prof. personal qualities) ^∗^16 variables of interpersonal interaction (8 dimensions) ^∗^prof. personal qualities: 3 scales (*knowledge, skills, personal qualities*) = 26 items	Interpersonal	Preferences about PETs’ char.*hyper-affiliating pers. = highest degree of manifestation; 2nd = *autocratic pers.*; lowest two = *humiliated* and *suspicious* pers. → PETs should be *benevolent, cooperative, helpful, showing empathy, strict* and *uncompromising enough* in organization and control during PE classes ^∗^gender: no diff. ^∗^age: diff. → desire for communication, understanding, cooperation with PET increases with stud.’ age Attitudes toward PETs’ prof. personal qualities→ PETs should be interested in stud.’ problems, maintain a closer interpersonal distance, socialize, advise, support, help ^∗^age diff. → 5th class stud. place higher imp. on PETs’ personal qualities than 7th class stud. ^∗^gender diff. → boys place less imp. on PETs’ skills ^∗^sports participation diff. → active kids place higher imp. on PETs’ personal qualities than kids not engaged in sports
Szczepanski (2012) Poland	^∗^cross-sec./quant. ^∗^312 PETs and 600 OSTs – prim. and sec. schools	To analyze differences reg. opinions on distinguishing char. of PETs perceived by PETs themselves and by OSTs	quest.: self-dev. (NN) ^∗^13 examined attributes - social distinguishing features (in PETs opinion) – self- and peer assessment	Trait psychological	^∗^PETs assess pos. image attributes (e.g., *cheerful lifestyle, O, honesty, immediacy, pos. thinking*) higher than OSTs ^∗^PETs proclaim *organizational ability, dynamic actions* and *cheerful pers.* distinguishing char./OSTs proclaim *PETs’ outfit, dynamic actions, cheerful pers.*
Voll (2006) Germany	^∗^cross-sec./quant. ^∗^976 stud. – year 8–10 – sec. schools	To examine stud.’ expectations toward their PET and to create a competence profile of an effective PET and examine diff. reg. stud.’ grade, school environment, school level, gender	quest.: self-dev. (NN) concerning PETs’ prof. competence/skills and char. (pers.: *fairness, understanding, partner, role model, assertiveness*)	Trait psychological	Competence profile of stud. needs oriented PET: *prof. expertise; great repertory; sporty; empathy; methodical-didactical skills; pedagogical charm; autogenesis companion; sport ethos; creator; sensitivity for needs* ^∗^several diff. mostly betw. grade 8 and 9, urban and rural schools, sec. and vocational schools; only diff. reg. gender for *responding to stud.* → more imp. for m.
Zalech (2011a) Poland	^∗^cross-sec./quant. ^∗^763 stud. (279 m.) – 2 senior high schools	To determine what features of PETs are most undesirable according to high school stud. and indicate if gender, grade or school affect selection of individual features	self-dev. (NN) diagnostic survey – quest. technique (semi open) ^∗^participants identify 3 char. a PET should not have ^∗^similar to Zalech (2011b)	Trait psychological	^∗^most undesirable features: quick temper (65%); severity (50%); unreliability/moodiness (37/34%) ^∗^gender and grade: various 2nd and 3rd order interactions → e.g., girls in grade I chose *strict* more often than boys; boys were more displeased at the PET’s *indecision* (independent of grade) → boys e.g., indicate *submissive* and *indulgence*; girls e.g., *being moody* and *quick-tempered* as undesirable features ^∗^school: no diff.
Zalech (2011b) Poland	^∗^cross-sec./quant. ^∗^744 stud. (273 m.) – 1st, 2nd, 3rd year – comprehensive upper-sec. school	To define what pers. traits, according to upper-sec. stud. are most significant in a PET and indicate if school, gender, year differentiate the choices	Diagnostic survey – quest. technique (semi open); self-dev. (NN) ^∗^participants identify 3 distinguishing pos. traits a PET should have → selection from 12 diff. adj. plus option to add 1 feature	Trait psychological	^∗^top 4 no gender diff.: *understanding* (53.5%), *fairness* (47.3%), *patience* (39.3%), *sense of humor* (39.3%); only order differs ^∗^least indicated: *caring* (6.1%)/*other traits* (3.5%) ^∗^variable interdependency (2nd and 3rd order interactions)
Zalech and Rutkowska (2014) Poland	^∗^cross-sec./quant. ^∗^22 PETs, 22 OSTs, 22 final-year stud. – upper-sec. school	To get to know the image of PETs seen by themselves and compare it with school community’s perception	quest.: *ACL-37* (Gough and Heilbrun, 2012) ^∗^300 adj. ^∗^participants choose fitting adj.	Trait psychological (Gough and Heilbrun)	^∗^diff. betw. PETs’ & others’ view → PETs perceive themselves in a more pos. manner (mark more favorable than non-favorable adj.) → 2 adj. most frequently associated with image of PETs by all groups: *active* and *energetic* → 6 most selected adj. (*open-minded, willing to cooperate, active, healthy, hard-working, skillful*) all pos. connoted

*Study Design/Method Sample: cross-sec., cross-sectional; m., male; OST, Other Subject Teacher; PET, Physical Education Teacher; prim., primary; priv., private; quant., quantitative; sec., secondary; stud., students; uni., university. Aim: betw., between; char., characteristics; diff., difference(s); m., male; OST, Other Subject Teacher; pers., personality; PET, Physical Education Teacher; PPC, Professional Personality Competence; priv., private; prof., profession(al); reg., regarding; relations., relationship(s); sec., secondary; stud., student(s). Personality Inventory: adj., adjectives; char., characteristics; diff., differen(t)ce(s); imp., importan(t)ce; pers., personality; PET, Physical Education Teacher; pos., positive(ly); prof., profession(al); quest., questionnaire; self-dev., self-developed; stud., student(s); subc., subcomponent(s); O, Openness; C, Conscientiousness; E, Extraversion; A, Agreeableness; N, Neuroticism. Main Results: adj., adjectives; betw., between; char., characteristics; corr., correlation(s); diff., differen(t)ce(s); f., female; imp., importan(t)ce; m., male; OST, Other Subject Teacher; PE, physical education; neg., negative; pers., personality; PET, Physical Education Teacher; pos., positive(ly); priv., private; prof., profession(al); PPC, Professional Personality Competence; relations., relationship(s); stud., student(s); subc., subcomponent(s); subs., subscale(s); O, Openness; C, Conscientiousness; E, Extraversion; A, Agreeableness; N, Neuroticism.*

### Stage 5: Collating, Summarizing and Reporting Results

We followed Arksey and O’Malley’s (2005) suggestion and divided this part into two different approaches of presenting the charted information: (1) Numerically analyzing the studies’ framework conditions and design and (2) Organizing the literature thematically.

## Results

Figure 1 documents the search and reference selection process. The initial search yielded 2316 hits: Education Source (*N* = 166), ERIC (*N* = 65), PsychARTICLES (*N* = 22), PsycINFO (*N* = 77), PSYNDEX (*N* = 24), PubMed (*N* = 18), Scopus (*N* = 462), SocINDEX (*N* = 70), SPOLIT (*N* = 1148), SPORTDiscus (*N* = 209), Web of Science (*N* = 55). After removing 270 duplicates, 2046 titles were screened. Six hundred sixty-three references did not meet the inclusion criteria and were excluded. Consequently, 1383 abstracts were screened. Seventy-eight abstracts met all inclusion criteria. The corresponding full-texts were purchased and screened. In this process, 49 studies were excluded, concluding with 29 studies. Three additional studies resulted from update search one.

Fifteen out of these 32 studies were published between 1958 and 1990 (including). No study was published between 1991 and 2005. Seventeen studies were published between 2006 and 2016. Studies before 1991 differed from studies after 2005 regarding the underlying personality understanding (following various theories, e.g., human needs theory, interpersonal theory, situational theory, behavioral theory, trait theory) and consequently assessment methods [e.g., *Edwards Personal Preference Scale* (Edwards, 1959) or *California Psychological Inventory* (Gough, 1957)]. Studies from 2006 onward mostly relied on other, newer personality understandings, as recognized personality concepts as well as assessment instruments emerged in the late 1980s and subsequent years [e.g., emergence of Costa and McCrae’s work and the publication of the first version of the *NEO-PI* (Costa and McCrae, 1985) or advancement of Holland’s *Self Directed Search* assessing vocational interests (Holland, 1994)]. Due to this gap in the literature and the mentioned content-related considerations, a comprehensive thematic presentation was exclusively done for studies published after 2005. However, in order to also give an overview of the older studies, we included the data and results of the 15 studies published between 1958 and 1990 in the supplementary section of this paper (see Supplementary Table 1). In the additional analyses step of the 17 included studies we deliberately checked not only for publications as from 2006 but also for publications in the years between 1991 and 2005. This process resulted in further six studies – all published later than 2005. In total, 23 studies were included in our final review. Table 1 provides a summary of the 23 finally included studies.

### Framework Conditions and Study Design

Sixteen different first authors published the 23 included studies, 15 thereof in the last 5 years. Ten studies could be allocated to the Middle East (including India) (Hosein Razavi et al., 2012; Demir, 2014, 2015a,b,c, 2016; Mantu and Montu, 2014; Arbabisarjou et al., 2016; Hassan et al., 2016; Maryam et al., 2017), eight to Eastern Europe (Brudnik, 2007, 2010; Zalech, 2011a, b; Szczepanski, 2012; Zalech and Rutkowska, 2014; Georgiev, 2016; Makhmutova et al., 2017), four to Western/Northern Europe (Brandl-Bredenbeck, 2006; Voll, 2006; Lauritsalo et al., 2015; Senn et al., 2017) and one to North America (García-Villanueva et al., 2017). All studies followed a cross-sectional study design. Twenty-two studies chose a quantitative, one study (Lauritsalo et al., 2015) a qualitative approach. Test persons were either PETs themselves (*N* = 14), teachers of other subjects (in the following abbreviated as OST = other subject teacher) (*N* = 5) or students (*N* = 10) evaluating PETs’ personality from an external view. Sample size varied between 20 and 312 for PETs, 20 and 600 for OSTs, 22 and 8863 for students. In order to assess personality the included studies used 19 different inventories – seven of which being well-recognized as personality inventories [*NEO-FFI* (McCrae and Costa, 2004); *NEO-PI-R* (Costa and McCrae, 1992); *SDS* Polish Version (Holland, 1997; Lacala et al., 2002); *EPQR-S* (Eysenck et al., 1985; Pourghaz et al., 2016); *ACL* (Gough and Heilbrun, 2012); *16PF* Form C of Russian version (Fetiskin et al., 2002) adapted from (Cattell et al., 1993); *Test of T Leary* (Leary, 2004) (*N* = 8)]. Six studies each either made use of less-recognized inventories or designed their own questionnaire according to the study’s needs.

### Personality Understanding, Research Questions and Results

As research questions of the analyzed studies are diverse, the presentation of the underlying personality understanding, research questions and results will be divided into three thematically coherent categories: (*I) The PET’s personality* – studies with their main focus explicitly on the identification of the PET’s personality (*N* = 3); (*II) The PET’s personality and correlates* – studies examining the PET’s personality in relation to another variable (*N* = 9); (*III) The PET’s personality from an external view* – studies interested in a non-PET view on the PET’s personality (*N* = 11). Within the categories according to the formulated three foci of the review’s research question, the studies’ underlying personality understanding together with their research questions and the studies’ results will be presented separately.

#### The PET’s Personality

In this category researchers are explicitly interested in the PET’s personality. In all three studies (Mantu and Montu, 2014; Hassan et al., 2016; García-Villanueva et al., 2017) personality is approached as universal and comprehensive. Mantu and Montu (2014) and Hassan et al. (2016) both intend to compare the personality characteristics of PETs with those of OSTs. They follow a trait psychological approach of personality. García-Villanueva et al.’s (2017) study follows a special understanding of the PET’s personality in the subject area of gender studies. The study’s primary aim is to analyze differences regarding sex, age and marital status among PETs in the four gender-related scales (*masculinity*, *femininity*, *machismo*, *submission*) considering personality characteristics.

Mantu and Montu (2014) conclude that there are no significant differences between the personality factors of PETs and OSTs considering the overall score. Solely when analyzing the individual factors, Mantu and Montu (2014) state that PETs are more *extraverted* than OSTs. Hassan et al. (2016) do not find any statistically significant differences in the individual *Big Five* factors of PETs and OSTs – *extraversion* and *conscientiousness* are most strongly pronounced in both teacher groups. García-Villanueva et al. (2017) do not find any statistically significant differences in the relationships of the four gender-related personality scales and the variables sex, age and marital status.

#### The PET’s Personality and Correlates

The nine studies that are assigned to this category state the relationship between the PET’s personality and one or more correlates as their main objective. Three of these studies examine the relationship between the PET’s personality and burnout (Brudnik, 2010; Makhmutova et al., 2017; Maryam et al., 2017). Brudnik (2010) speaks of vocational personality, Makhmutova et al. (2017) of personality development within a trait psychological approach and Maryam et al. (2017) of personality traits in general. All three also assess additional aspects such as self-concept or mental health. Demir’s three studies in this category (Demir, 2014, 2015a,b) aim at examining the relationship between PETs’ personality traits and their sports branches (e.g., football, swimming, gymnastics) and sports type (team sports vs. individual sports). In two of the studies (Demir, 2014, 2015a) he also examines the PET’s gender, school type (private vs. public schools) [in 2015a also school level (secondary vs. high schools)] and years of service in relation to the PET’s personality. In two studies (Demir, 2014, 2015b) he follows a trait psychological understanding of personality. In his third study (Demir, 2015a) he speaks of professional personality competence and by this identifies the PET’s vocational personality. Brudnik (2007) follows Holland’s tradition which understands vocational interests as personality characteristics and therefore also establishes a work-related peculiarity of personality. Similar to Demir’s studies she examines the relationship between the PET’s vocational personality and gender, type of school and work environment. Hosein Razavi et al. (2012) and Arbabisarjou et al. (2016) examine the relationship between PET’s personality traits and students’ individual and social behavior or the entrepreneurial organizational culture, respectively. Arbabisarjou et al. (2016) follow a trait psychological understanding of personality whereas Hosein Razavi et al. (2012) speak of entrepreneurial personality characteristics and therefore follow a vocational approach.

Studies examining the PET’s personality in relation to burnout all focus on different analyses and therefore conclude with multifaceted results. Brudnik (2010) finds that PET’s gender is related to the burnout path – male PETs burnout following a particular path whereas female PETs burnout less uniformly. Further, Brudnik (2010) finds out that self-efficacy – which is often seen as part of the personality – serves as preventive factor of burnout for OSTs. The degree of the PET’s personality matching the profession (*SDS*; Holland, 1994) only slightly affects the burnout path. Makhmutova et al. (2017) highlight the fact that PETs scoring higher on the intellectual development level [*Scale B of Cattell’s 16PF* (Cattell et al., 1993) – reasoning] are less satisfied with their work conditions and by this more likely to burnout. Qualified PETs – graduated in PE – exhibit significantly higher rates in the subscales *reasoning* and *emotional stability* compared to non-qualified PETs (Makhmutova et al., 2017). According to Maryam et al. (2017) when considering the *Big Five* personality factors only *neuroticism* shows a positive correlation with PETs’ burnout development (via the burnout indicator *emotional exhaustion*).

Demir’s results in his methodologically similar studies are contradictory. In his study from Demir (2015b) he does not detect a correlation between the PET’s sports type (individual sports vs. team sports) but examines significant correlations between two personality sub dimensions (*emotional stability* and c*ompatibility*) and the PET’s sports branches. Racket sports and handball players are *emotionally more stable* than gymnasts. Basketball and defense sport players are more *compatible* than swimmers. In his earlier study from 2014 he does not find any significant differences between PETs’ personality and their sports branch, sports type or the other examined correlates (gender, years of service, school type). In his study from 2015a he detects differences in PET’s vocational personality regarding the school type and school level the PETs teach in, but not regarding their gender. PETs in private schools and high schools are more competent regarding their professional personality than their colleagues in public schools or secondary schools. Brudnik (2007) finds a difference between male and female PETs’ vocational personality code. *Social, Investigative, Artistic (SIA)* summarizes females’ vocational personality whereas *Social, Realistic, Enterprising (SRE*) is the male equivalent. She cannot show a relationship between the vocational personality and work environment or school type.

Hosein Razavi et al. (2012) and Arbabisarjou et al. (2016) both find significant correlations between at least some personality sub dimensions and their examined correlates. Arbabisarjou et al. (2016) only report correlations without mentioning directions of these. The sub dimensions *extraversion* and *openness* of the PET’s personality have a significant relationship with the students’ beliefs and activities (Arbabisarjou et al., 2016). Hosein Razavi et al. (2012) find that three of the six components of entrepreneurial organizational culture (*creative innovation*, *cooperation* and *tolerance of creative talents*) obtain a negative correlation and two components (*organizational risk-taking* and *open communication*) a positive correlation with the PET’s entrepreneurial personality characteristics.

#### The PET’s Personality From an External View

Category III consists of studies that aim at receiving an external view on the PET’s personality. The eleven studies in this category examine OSTs and students as members of the school community. The category can be divided into three thematically coherent groups: *(1) Studies generally describing the PET’s personality*; *(2) Studies obtaining attitudes of/opinions toward PET’s personality; (3) Studies describing “the ideal PET.”* Three studies each can be assigned to group (1) (Zalech and Rutkowska, 2014; Lauritsalo et al., 2015; Senn et al., 2017) and (3) (Voll, 2006; Zalech, 2011a, b). Five studies belong to group (2) (Brandl-Bredenbeck, 2006; Szczepanski, 2012; Demir, 2015c, 2016; Georgiev, 2016).

In group (1), Zalech and Rutkowska (2014) compare the image of the PET from the PET’s own perspective with students’ and colleagues’ descriptions. Senn et al. (2017) are interested in the relationship of PET’s personality characteristics with students’ motivation in PE, solely considering the students’ view. Lauritsalo et al. (2015) aim at collecting an unbiased overview of students’ attitudes toward school PE in Finland by collecting messages from chat protocols in internet discussion forums. Lauritsalo et al. (2015) do not mention the PET’s personality in their aim but as an outcome factor – together with the PET’s behavior. Lauritsalo et al. (2015) and Senn et al. (2017) closely associate PET’s personality with behavior whereas Zalech and Rutkowska (2014) follow a trait psychological approach. In group (2) Brandl-Bredenbeck (2006), Demir (2015c, 2016), and Georgiev (2016) aim at obtaining the students’ attitudes toward their PETs. Georgiev (2016) follows Leary’s (Leary, 2004) theory of interpersonal interaction in order to assess personality. Demir (2015c, 2016) speaks of teachers’ professional personality and therefore follows a vocational personality standpoint. Brandl-Bredenbeck (2006) speaks of personality in general closely related to behavior and supplements this general approach by examining the PET’s care as additional personality aspect. Szczepanski (2012) also asks for opinions on the PET’s personality but compares PETs’ and OSTs’ views, explicitly speaking of image or identity and therefore being in line with trait theory. The authors in group (3) – Voll (2006) and Zalech (2011a, b) – explicitly ask for the ideal (or not ideal, Zalech, 2011a) PET and all follow a trait psychological approach of personality.

In Zalech and Rutkowska’s (2014) study PETs evaluate themselves more positively than their colleagues (OSTs) or students. OSTs and students describe the PETs as e.g., less patient, less hard-working and less intellectual compared to PETs’ views. In total, PETs mark more favorable than non-favorable adjectives when describing their personality with a choice of given adjectives. The three groups are in agreement with each other regarding the most characteristic identity attributes of PETs – all mentioning active and energetic (Zalech and Rutkowska, 2014). Senn et al. (2017) detect differences regarding the students’ gender and sportiness when assessing the role of the PET’s personality for their motivation in PE. Girls put more emphasis on the skill that the PET realizes their fears and sporty kids choose different attributes as important for their motivation (e.g., achievement orientation and strict grading) compared to less sporty kids. Lauritsalo et al. (2015) detect more negative, not empathetic statements regarding the PET’s personality than positive ones. Students describe PETs as not supportive, accompanied by strong feelings of compulsion and humiliation (Lauritsalo et al., 2015). In total, in this study 40 percent of the analyzed messages contain statements regarding the PET’s personality or behavior – making this facet the dominant outcome variable.

Georgiev (2016) finds out that younger students put more emphasis on PETs’ caring behavior and interest in their problems than older ones. The desire for communication, understanding and cooperation with the PET increases with the students’ age. In Szczepanski’s (2012) study, PETs rate positive image attributes (e.g., *cheerful lifestyle*, *openness*, *honesty*, *immediacy*, and *positive thinking*) of themselves higher than their colleagues (OSTs). The biggest difference occurs for the personality characteristic *organizational ability*. Considering the PETs’ opinion, the top three characteristics, which distinguish them from their colleagues, are *organizational ability*, *dynamic actions* and *cheerful personality*. OSTs mention the PETs’ *clothing style* as the strongest distinguishing feature followed by *dynamic actions* and *cheerful personality*. Demir (2015c, 2016) is again represented with two studies in this category, both obtaining students’ attitudes toward their PET’s professional personality competence. Demir (2016) finds significant gender differences for one subcomponent (*motivational interaction*) only – girls being more satisfied with their PET’s *motivational interaction* than boys. In his earlier study (Demir, 2015c) he finds differences for three subcomponents – *motivational interaction*, *professional enthusiasm/dedication*, and *reflective interaction*, – girls being more satisfied with the first two and boys with the last subcomponent. Demir (2015c) also highlights the fact that younger students – grade nine and ten students - and students of private schools are more satisfied with their PET’s professional personality competence compared to grade 11 students and counterparts in public schools (school type differences also in Demir, 2016). In Brandl-Bredenbeck’s (2006) study PETs are perceived as *self-confident*, *caring* and *friendly* by their students. In total, he speaks of a positive evaluation. Younger PETs receive a better evaluation than older PETs.

Zalech (2011b) detects *understanding*, *fairness*, *patience*, and *sense of humor* as the four most desired attributes of a PET. He does not find any differences regarding the students’ gender. In his study asking for the most undesired features of a PET (2011a) though the choice differs significantly between girls and boys. Boys e.g., indicate *submissive* and *indulgence* as undesirable features, whereas girls, e.g., indicate *being moody* as well as *quick-tempered.* Zalech (2011b) also finds a second-order interaction between gender and grade of students with girls in grade one for example choosing *strict* significantly more often as most undesired feature than boys in the same grade. Schools though do not have a significant impact on the choice. Voll (2006) finds out that students in grade eight generally put more emphasis on all examined personality characteristics (*fairness, understanding, being a partner, being a role model, assertiveness*) than their counterparts in grade nine. Voll (2006) also detects differences regarding school type or level. Students in urban schools, e.g., put more emphasis on the PET’s *fairness* than their counterparts in rural schools. Further, students in vocational schools put more emphasis on the PET’s *assertiveness* than Realschule (German middle school) students.

## Discussion

Our review aimed at summarizing the status of research concerning the personality of the PET. After the screening process 23 studies were included. The chosen methodology of a scoping review – following a rather broad approach with soft exclusion criteria – tried to make sure that all studies coming within our aim (*Summarizing empirical studies – their underlying personality understanding, research questions and results – considering the personality of the PET*) were included in the final review. Other scoping studies in our field that can be considered as balanced, analyze a similar number of studies [e.g., Richards et al., 2017 (*N* = 20); Sperka and Enright, 2017 (*N* = 31); Robinson, 2018 (*N* = 30); Killian et al., 2019 (*N* = 24)] and conclude with promising results, partly providing practical implications and indications for future research. Due to the studies’ heterogeneity, results are hard to synthesize and compare among each other or with our results. All 23 in our review analyzed studies were cross-sectional, all but one quantitative. The underlying personality understanding but also the research questions and results of the included studies varied enormously and by this supported the assumption that the research field is wide and construed differently.

### Discussion of Framework Conditions and Study Design

Twenty of the 23 included studies were published in 2010 or later – fifteen thereof between 2014 and 2017. Therefore, we can speak of an increasing research interest with regard to the PET’s personality in the last years. This might be caused by Hattie’s (2009) world-renowned meta-analysis stating the teacher’s personality as one essential factor of successful learning. Considering the origin of the included studies, it is surprising that 18 studies originate from the Middle East or Eastern Europe. This might be due to political changes at around this time or probably in consequence of the PISA study’s results in 2000 and subsequent survey times. The studies’ sample size varied distinctly. For eight studies it seems difficult to generalize findings due to small sample sizes (Mantu and Montu, 2014; Demir, 2015a, b; Arbabisarjou et al., 2016; Georgiev, 2016; Hassan et al., 2016; García-Villanueva et al., 2017; Makhmutova et al., 2017).

### Discussion of Personality Understanding, Research Questions and Results

The amount of different inventories used to assess personality (*N* = 19) emphasizes the assumption of a prevailing diversity among the different approaches to personality. Only five studies (Demir, 2014, 2015b; Arbabisarjou et al., 2016; Hassan et al., 2016; Maryam et al., 2017) used a *Five Factor* inventory and by this follow the *Five Factor* structure of personality (Costa and McCrae, 1999). Considering the fact that in general – not teaching context specific – personality research the *Five Factor* understanding of personality predominates the research area (John et al., 2008; Göncz, 2017), this number here can be considered rather small. Also only six studies followed a vocational approach of personality. This number was expected to be greater due to the chosen profession specific context.

In the following, the studies’ research questions and results will be discussed separately, following the same three-part structure as before.

#### The PET’s Personality

Interestingly, the personality between PETs and OSTs does not differ considerably according to the two studies approaching this question (Mantu and Montu, 2014; Hassan et al., 2016). Solely considering the factor *extraversion*, the PETs score significantly higher than OSTs, signifying that they are more extraverted. This becomes interesting and relevant when considering Kim et al.’s (2019) results that out of the *Big Five* domains, *extraversion* obtained the strongest association with the teacher’s effectiveness and by this can be seen as favorable characteristic. Mantu and Montu’s (2014) result that PETs are more *extraverted* than OSTs hinders that they particularly can positively influence their students’ learning process. Due to the fact that only two of the included studies dealt with this topic, the implications have to be treated with caution though. García-Villanueva et al.’s (2017) study clearly stands out when comparing personality understanding and research questions. The content of this study can be considered as stand-alone among the others. Also in general educational research we could not find an equivalent study (*inter alia* Göncz, 2017).

#### The PET’s Personality and Correlates

PETs’ burnout risk is clearly the dominant correlate among the included studies. Considering the publication dates of the included studies in our review, the fact that it is still only examined in three studies is in line with burnout research’s development in the last decade. Teacher burnout research gained popularity at the turn of the millennium (Krause, 2003). In this time, as a result of empirical investigations, the widely known assumption that teachers obtain stress and strain levels higher than workers in other professions emerged (Maslach et al., 1996; Schaufeli and Enzmann, 1998; Schaarschmidt, 2004, 2005). Nowadays though after a decade of intensive research on this topic, work-related well-being is often approached from a positive perspective considering resources instead of demands and by this e.g., examining positive motivational processes and psychological states such as work engagement instead of burnout (Schaufeli et al., 2009). This is in line with psychology’s orientation toward a *Positive Psychology* starting around the turn of the millennium (Schaufeli et al., 2009). Interestingly, the few included studies on this topic in our review, even though published later than 2006 follow the traditional understanding of burnout and conclude with a relationship between PETs’ personality factors and their burnout level. As the results have shown, the amount and exact manifestation is unclear though. The orientation toward burnout might be explicable with our review’s focus examining PET’s personality. This orientation and therefore the relationship between teacher burnout and personality is also a common research topic in recent general educational research, especially when examining indicators for professional success. Cramer and Binder (2015) and Kim et al. (2019) examined the relationship between *Big Five* personality characteristics and burnout among teachers in general and conclude with similar results: high scores on *neuroticism* solidly indicate an increased burnout risk and low scores on *extraversion* and *conscientiousness* seem to indicate at least partly a reduced burnout risk. This is in line with Maryam et al.’s (2017) results – the only study in our review that analyses the relationship between PET burnout and *Big Five* personality characteristics. In comparison to studies considering teachers in general, the topic seems to be rather understudied for PETs. Research considering the PET’s stress though – without linking it to personality and rather connecting it to their health – has gained popularity in recent years. Brandt (2019) highlights this fact in his dissertation summarizing quantitative and qualitative studies examining the PET’s health. He concludes that PETs obtain rather high stress levels and are health wise more vulnerable than OSTs.

Demir (2014, 2015a,b) concentrates his research on the relationship between the PET’s personality and the PETs’ sporting practice – a focus area which does not receive a lot of attention in previous studies. It becomes interesting in the discussion on how much practical education PETs should receive at university, how comprehensive this should be and concomitant which sporting competencies should be condition for entering a teaching degree. In previous research it was only the overall picture of the PET’s sportiness (Messing, 1979) that received attention whereas Demir (2015b) goes into detail and differentiates in terms of the particular practiced sport – sorted by branch and type. Due to the fact that his results are contradictory this approach does not raise hope for practical implications though.

Only one study (Arbabisarjou et al., 2016) examines the relationship of the PET’s personality and students’ actual behavior in the lesson and by this links the PET’s personality to student participation and motivation in PE. This link is common in general educational research. Kunter et al. (2013b) for example revealed positive effects of the teacher’s personality (in this particular case enthusiasm) on instructional quality and by this on student outcomes, such as motivation or achievement. Wayne and Youngs (2003) pursued this relationship in a literature review also concluding with the fact that certain teacher characteristics foster student achievement. Arbabisarjou et al.’s (2016) results are especially interesting when following educational research’s assumption that the teacher influences student motivation and learning success (Hattie, 2009; Erpic, 2013; Kim et al., 2019). Considering Arbabisarjou et al.’s (2016) results, the personality factors *extraversion* and *openness* should therefore receive attention when considering student participation and motivation in PE, e.g., in teacher education or lesson planning. Arbabisarjou et al. (2016) raise the awareness for the right amount of interpersonal relations, creativity and flexibility when teaching. Even though the variability of the personality characteristics is rather small, knowing the individual manifestation, such as being overly *extraverted* and *open*, can help teachers in order to motivate students when deliberately playing to their own strengths. Conversely, less *extraverted* or less *open* teachers need to be presented with or find other strategies in order to ensure their students’ motivation. Senn et al.’s (2017) study (category three) runs in a similar direction but only works with one variable (students’ attitudes). Other than that, to the best of our knowledge, this explicit and interesting relationship has not been examined in PE context so far.

Brudnik (2007) and Demir (2015a) both following predominantly a vocational approach, conclude with contradictory results – no gender differences regarding PET’s vocational personality in Demir’s study but in Brudnik’s; no differences regarding context factors in Brudnik’s study but in Demir’s. This might be explicable with their interpretation of vocational personality. Brudnik (2007) follows Holland’s (1994) understanding asking for *preferred activities*, *possessed skills* and *professional preferences* whereas Demir’s (2015a) scale includes the self-evaluation of *professional enthusiasm*, *respect for human dignity* and *interactional components* (*reflective* and *stimulating*) and by this partly follows an interactive approach within the vocational understanding. Demir’s (2015a) decision to ascertain enthusiasm is again in line with modern general educational research’s understanding of the teacher’s professional competence (e.g., Baumert and Kunter, 2011) including a broad understanding of the term personality. Teacher enthusiasm in general educational research is often examined in relation to student outcomes such as motivation. Keller et al. (2013) suggested a *personal trait like enthusiasm understanding* within an integrated model of teacher enthusiasm and by this highlighted the relationship to and importance of personality characteristics.

#### The PET’s Personality From an External View

With 11 studies in this category, examining an external view of the PET’s personality can clearly be seen as a methodological peculiarity among the included studies. Connelly and Hulsheger (2012) were able to show that external observers have a clearer view on a person’s personality and are therefore able to provide a certain depth of personality information. Further, Dinger et al. (2014) comparing self and observer reports of personality functioning conclude that the combination of both views was most efficient and should therefore be considered in future research. Observer reports certainly add essential information and offer possibilities for incorporating bordering approaches upon personality.

Brandl-Bredenbeck (2006) incorporates the PET’s care estimated by students as part of the PET’s personality. This understanding borders upon Self-Determination-Theory (*SDT*) (Deci and Ryan, 2002) – considering the PET’s care as part of *SDT*’s factor relatedness. Interestingly, research focusing on the teacher’s care – often in relation to student engagement (Nie and Lau, 2009) or student motivation (Thompson, 2010; Bieg et al., 2011) – is mostly located in general educational research. Especially in PE context though where PET’s relationship closeness to students automatically receives importance, caring aspects seem to be influential. Brandl-Bredenbeck’s (2006) approach of examining PET’s care could be interesting, especially for researchers linking PET’s personality with students’ personality and further with their learning motivation.

Five studies aim at receiving attitudes/opinions toward the PET’s (personality) which is also a common research aim in general educational research (Göncz, 2017). Interesting is also group three’s focus – *the ideal PET*. Receiving attitudes/opinions toward the teacher and looking upon the ideal teacher are also visible strategies in the configuration of prevailing didactical concepts. Concretizations among these are e.g., obtaining students’ attitudes toward their teacher as basis for further decisions when planning lessons or when teaching (e.g., making use of the methodology *student reflection* in order to influence students affectively, Cavilla, 2017). Additionally the focus area raises the predominant question if there is such a thing as the ideal teacher or the good and desired educator personality (Weinert and Helmke, 1996). Studies in category three in our review acknowledge the fact that students are valuable evaluators of their PE lessons (e.g., Brandl-Bredenbeck, 2006; Voll, 2006) and by this also their PET. They deliberately ask for desired or undesired character features (e.g., Zalech, 2011a, b) and believe that this information and empirical evidence can serve as a base for student-centered and adapted teaching. Amongst this content-related salience, category three comprises the only qualitative study (Lauritsalo et al., 2015) which follows a rather modern and in this research field unprecedented approach – screening internet chat forums. The approach itself certainly is exciting as it does not face typical problems that occur in questionnaire surveys, e.g., limited options to answer or drifting to the center when answering and therefore produces “relatively authentic natural data” (Holtz et al., 2012). It is necessary though to check if adolescents in chat forums really venture their personal opinions or the desired opinion of their friends.

The results regarding the PET’s appearance – considered here as part of their personality (e.g., in Szczepanski, 2012; Zalech and Rutkowska, 2014) – resemble the common belief that PETs represent special personalities and can be distinguished from OSTs. It opens up questions and ideas for career advice for instance. Interestingly, the PETs evaluate themselves in a more positive light than their colleagues. This might be due to a generally higher evaluation of oneself by e.g., faking answers in order to appear socially better (Sjöberg, 2015) or because PETs in general possibly come off differently compared to OSTs such as Mantu and Montu’s (2014) results hint for the personality factor *extraversion*.

Overall, it is noticeable that when examining students, most studies also distinguish between the students’ gender, the grade they are in and the school they attend. Senn et al. (2017), connecting the PET’s personality to students’ motivation, directly ask for motivation enhancing personality characteristics and detect gender and age differences between girls’ and boys’ perception. In addition, girls and boys in Voll’s (2006), Zalech’s (2011b), and Demir’s (2015c) studies assess different PET personality characteristics as important and desirable. Consequently, when teaching single-sex groups of students it might be easier for the PET to satisfy the students’ expectations and perform suitable for the taught group. In line with previous general educational research (e.g., Samdal et al., 1998) is the fact that younger students seem to be more satisfied with their teacher. Even though younger students compared to older students in general tend to be more satisfied with school and the teacher (Samdal et al., 1998), the studies’ results could predict the need for raising the awareness of the topic *PET personality* especially in the area of secondary school teaching and concomitant teacher education as elder students seem to be more particular. Knowing their personality characteristics could therefore be beneficial for PETs in order to succeed when teaching this age group. It allows PETs again to play to their own strengths or deliberately focus on different motivational approaches detached from their personality. Another dominant result covers differences regarding the visited school (type and level) – both on the teacher and the student side. School type (private vs. public) but also school level (e.g., middle school, higher level secondary school, vocational school) affect the evaluation of the PET’s personality (e.g., Voll, 2006; Demir, 2015c). This presages the possibility of a voluntary personality examination serving as assistance in the decision for a school-specific teaching degree program. Some states in Germany (Nordrhein-Westfalen, Baden-Württemberg and Rheinland-Pfalz) and the teachers colleges in Austria e.g., use CCT (Bergmann et al., n.d.) a web-based consulting tool, including the examination of personality characteristics. This tool serves as assistance in the decision process for students entering a teaching degree program.

Lauritsalo et al.’s (2015) study is the only one among the included studies that in general speaks of a rather negative image the students assign their PETs. Again, the chosen methodology can affect the results as e.g., group pressure could have led to the dominance of negative statements. This might be due to the users’ tendency to make more extreme and more offensive statements on the internet (Williams et al., 2002). All other studies that examine the students’ image of the PET’s personality conclude with a positive picture.

## Implications

In total, the results reflect the included studies’ diverse methodological approaches and aims. This is also in line with general educational research’s findings concerning the topic *teacher personality*. Göncz’s (2017) five types of teacher personality studies – (1) Teacher typologies; (2) Studies of teachers’ desirable and undesirable features; (3) Studies of teachers’ professional behaviors and their influence on students; (4) Studies of teachers’ professional identities and (5) Studies of teacher personality within the framework of personality theories – can also be retrieved in our results. Type (1) *Teacher typologies* though is represented the least with only Brudnik (2007) speaking of teacher vocational personality codes and by this in the broadest sense also typologies. Even though not included in our review, Bräutigam (1999) can be seen as exemplary and popular study among PETs, examining students’ opinions concerning the *bad PET* and concluding with PET’s behavior typologies. He does not speak of personality, neither in his methodology nor in his outcomes and therefore was not included in our review, but the methodology of creating typologies and by this tangible results, seems promising and has obtained acceptance. Identifying typologies is a common and convenient approach especially when trying to derive practical implications and therefore should be considered in future research examining PETs’ personality.

Göncz’s (2017) type (2) *Studies of teachers’ desirable and undesirable features* mostly implies other-reports, in his review as well as in our review. Kim et al. (2018) highlight possibilities and strengths of other-reports in this research field specifically as well and concluded with stronger associations between other-reports of teacher personality and outcomes (teacher effectiveness and burnout) than self-reports. Other-reports as mentioned before therefore seem to be a promising approach when examining the PET’s personality and deriving practical implications.

Göncz (2017) addresses the partially low methodological quality in this field. We can support this assumption considering the included studies’ methodological quality in our review. The number of participants, e.g., is often even adduced by the authors themselves as limiting factor, reducing their study to a case study (e.g., Brudnik, 2010). Demir’s sample sizes vary enormously. He e.g., compares data from 1148 students from public schools with data from 273 students from private schools (Demir, 2016). In other studies the description of the undertaken methodological approach and the presentation of results are even unclear and partly contradictory and therefore have to be interpreted with caution (e.g., Hosein Razavi et al., 2012).

In total, we can speak of insufficient evidence in total and therefore suggest a cautious application of the aforementioned results and discussed issues, especially when considering the implication into teaching practice. We can align ourselves with Göncz (2017) when advising to follow the traditional personality models (e.g., *Five Factor* understanding) in order to ensure high methodological quality and a uniform foundation for educational research and valuable comparisons. Kim et al. (2018) focus specifically on the *Big Five* and conclude with valuable results for the evaluation of teaching. All *Big Five* domains except for *agreeableness* obtained a positive association with e.g., teacher effectiveness. They as well highlight the need for common, universal descriptors in teacher personality research and associated dissemination. This can especially be helpful for the abovementioned situations where PETs can play to their own strengths and make use of their individual personality configuration in order to teach successfully.

## Limitations

We decided to keep our understanding of personality as wide as possible in order to include all relevant studies and in order to answer the formulated research question. Therefore, the included studies had to actually measure personality as a variable or mention personality as an outcome. We acknowledge the fact that this procedure might have eliminated interesting studies that examine similar, related variables without mentioning personality explicitly. We also acknowledge the fact that by limiting our review to English and German publications – due to feasibility reasons – we might have lost relevant and interesting literature published in other languages.

## Conclusion

In conclusion, results of the included studies differ significantly, are partly contradictory and partially exhibit major methodological shortcomings. Considering the underlying personality understanding, most studies (*N* = 12) follow a trait psychological understanding of personality. Six studies follow a vocational and one study an interpersonal personality understanding. The remaining four studies’ underlying personality understanding is not concretely determinable but three out of the four studies are oriented toward an interactionist/behavioral view (see Table 1). The identification of these three prevailing orientations with the dominance of the *FFM* implies a rather consolidated orientation of the research field. Overall, this picture is congruent with general educational research’s orientation toward a mostly trait psychological understanding. Due to the fact that the vocational as well as the interactionist/behavioral approach yields interesting results we suggest following a rather wide approach of personality. Within this wide approach it is advisable though to also follow generally accepted approaches of personality in order to compare results and to facilitate the creation of practical implications. Alongside the idea of including various facets of personality in promising research, the compilation of different viewpoints, especially when aiming at the impact of the PET’s personality on student-related aspects, seems promising.

Considering examined correlates in relation to the PET’s personality, the two-part alignment prevalent in general education research mentioned in the introduction – personality in relation to student-related or teacher-related factors, mostly *success outcomes* – cannot be replicated in our review. Studies in our review mostly examine the relationship between the PET’s personality and correlates of sociodemographic nature (e.g., gender, age). The promising results in general educational research and the significance which general educational research and teacher competence models attribute to the teacher’s personality, leads to the conclusion that examining the PET’s personality in relation to the aforementioned *success outcomes* should receive more attention and therefore be considered in future research.

## Author Contributions

MS, AK, SS, and FM conceived and designed the study. MS and AK performed the literature search and study selection process. MS, SS, and AK performed the final analysis process. MS wrote the manuscript with substantial contributions from AK, SS, and FM. All authors approved the final version of the manuscript.

## Conflict of Interest

The authors declare that the research was conducted in the absence of any commercial or financial relationships that could be construed as a potential conflict of interest.

## References

[B1] Arbabisarjou A. Sourki M. S. Bonjar S. E. H. (2016). Students’ individual and social behaviors with physical education teachers’ personality. *Int. Educ. Stud.* 9 154–160.

[B2] Arksey H. O’Malley L. (2005). Scoping studies: towards a methodological framework. *Int. J. Soc. Res. Methodol.* 8 19–32. 10.1080/1364557032000119616 29722555

[B3] Bastian K. C. McCord D. M. Marks J. T. Carpenter D. (2017). A temperament for teaching? associations between personality traits and beginning teacher performance and retention. *AERA Open* 3 1–17. 10.1177/2332858416684764

[B4] Baumert J. Kunter M. (2011). “Das Kompetenzmodell von COACTIV,” in *Professionelle Kompetenz von Lehrkräften - Ergebnisse des Forschungsprogramms COACTIV*, eds Kunter M. Baumert J. Blum W. Klusmann U. Krauss S. Neubrand M. (Münster: Waxmann), 29–54.

[B5] Baumert J. Kunter M. (2013). “The COACTIV model of teachers’ professional competence,” in *Cognitive Activation in the Mathematics Classroom and Professional Competence of Teachers: Results from the COACTIV Project*, eds Kunter M. Baumert J. Blum W. Klusmann U. Krauss S. Neubrand M. (New York, NY: Springer), 25–48. 10.1007/978-1-4614-5149-5_2

[B6] Bergmann C. Brandstätter H. Demarle-Meusel H. Eder F. Kupka K. Mayr J. (n.d.). *CCT.* Available at: http://www.cct-austria.at/CCT/SetAudience (accessed October 31, 2019).

[B7] Bieg S. R. Backes S. Mittag W. (2011). The role of intrinsic motivation for teaching, teachers’ care and autonomy support in students’ self-determined motivation. *J. Educ. Res.* 1 122–140.

[B8] Brandl-Bredenbeck H. P. (2006). “Der/die Sportlehrer/in aus Sicht der Schüler/innen,” in *dvs Sektion Sportpädagogik: Zum Umgang mit Vielfalt als sportpädagogische Herausforderung*, eds Miethling W.-D. Krieger C. (Kiel: Czwalina).

[B9] Brandt C. B. (2019). *Prädiktoren für die Zugehörigkeit zum Mustertyp Gesundheit und Ansätze zum Verbleib und Dropout bei Sportlehrkräften zwischen 50 und 65 Jahren an allgemein bildenden Gymnasien.* Ph.D. thesis, University of Konstanz, Konstanz.

[B10] Bräutigam M. (1999). So schlecht ist er auch wieder nicht!” Erste Zugaenge auf die Frage nach dem “schlechten” Sportlehrer aus Schuelersicht. *Sportunterricht* 48 100–111.

[B11] Brettschneider W.-D. (2006). *DSB-Sprint-Studie: eine Untersuchung zur Situation des Schulsports.* Aachen: Meyer & Meyer.

[B12] Brudnik M. (2007). Vocational personality of a physical education teacher. *Hum. Mov.* 8 46–56.

[B13] Brudnik M. (2010). Macro-paths of burnout in physical education teachers and teachers of other general subjects. *Stud. Phys. Cult. Tour.* 17 353–365.

[B14] Buchanan T. Johnson J. A. Goldberg L. R. (2005). Implementing a five-factor personality inventory for use on the internet. *Eur. J. Psychol. Assess.* 21 115–127. 10.1027/1015-5759.21.2.115

[B15] Büyüknacar C. (2008). *The Study of the Relationship Between Emotional Competence and Professional Personality Traits of Anatolian and Science High School Teachers on the Basis of Students’ Perceptions (A Case Study in Gaziantep).* Master’ thesis, Gaziantep University, Turkey.

[B16] Cattell R. B. Cattell A. K. Cattell H. E. P. (1993). *16PF Fifth Edition Questionnaire.* Champaign, IL: Institute for Personality and Ability Testing.

[B17] Cavilla D. (2017). The effects of student reflection on academic performance and motivation. *SAGE Open* 7:215824401773379 10.1177/2158244017733790

[B18] Connelly B. S. Hulsheger U. R. (2012). A narrower scope or a clearer lens for personality? Examining sources of observers’ advantages over self-reports for predicting performance. *J. Pers.* 80 603–631. 10.1111/j.1467-6494.2011.00744.x 22091626

[B19] Costa P. T. McCrae R. R. (1985). *The NEO Personality Inventory Manual.* Odessa, FL: Psychological Assessment Resources.

[B20] Costa P. T. McCrae R. R. (1992). *Revised NEO Personality Inventory (NEO-PI-R) and NEO Five-Factor Inventory (NEO-FRFI) professional manual.* Odessa, FL: Psychological Assessment Resources.

[B21] Costa P. T. J. McCrae R. R. (1999). “A Five-Factor Theory of Personality,” in *Handbook of Personality: Theory and Research*, 2 Edn, eds Pervin L. A. John O. P. (New York, NY: Guilford), 139–153.

[B22] Cramer C. Binder K. (2015). Zusammenhänge von persönlichkeitsmerkmalen und beanspruchungserleben im lehramt. ein internationales systematisches review. *Z. Erziehwiss.* 18 101–123. 10.15496/publikation-11047

[B23] Deci E. L. Ryan R. M. (2002). *Handbook of Self-Determination Research.* Rochester, NY: University of Rochester Press.

[B24] Demir E. (2012). *Working in Secondary School Physical Education Teachers’ Professional Personality of Competence Perceptions of Assessment.* Istanbul: Phd Marmara University.

[B25] Demir E. (2014). *Evaluation of Personality Traits of Physical Education Teachers.* Saarbrücken: Lampert.

[B26] Demir E. (2015a). Assessment of Professional Personality Competence of Physical Education teachers working in Cannakale. *Pamukkale J. Sport Sci.* 6 17–32.

[B27] Demir E. (2015b). Evaluation of personality traits of physical education teachers working in secondary schools and high schools in çanakkale according to their sports branch. *US. China Educ. Rev. B* 5 27–34. 10.17265/2161-6248/2015.01.003

[B28] Demir E. (2015c). Students’ evaluation of professional personality competencies of physical education teachers working in high schools. *US China Educ. Rev. A* 5 149–157. 10.17265/2161-623x/2015.02.008

[B29] Demir E. (2016). Evaluation of Professional Personality Competence of Physical Education Teachers Working in Secondary Schools by Students. *J. Educ. Train. Stud.* 4 60–66. 10.11114/jets.v4i2.1116

[B30] Dinger U. Schauenburg H. Hörz S. Rentrop M. Komo-Lang M. Klinkerfuß M. (2014). Self-report and observer ratings of personality functioning: a study of the opd system. *J. Pers. Assess.* 96 220–225. 10.1080/00223891.2013.828065 24003849

[B31] Djigić G. Stojiljković S. Dosković M. (2014). Basic personality dimensions and teachers’ self-efficacy. *Procedia Soc. Behav. Sci.* 112 593–602. 10.1016/j.sbspro.2014.01.1206 15152849

[B32] Döhring V. Gissel M. (2016). *Sportunterricht Planen und Auswerten: ein Praxisbuch für Lehrende und Studierende.* Baltmannsweiler: Schneider-Verl. Hohengehren.

[B33] Dunkin M. J. Biddle B. J. (1974). *The Study of Teaching.* Holt: Rinehart & Winston.

[B34] Edwards A. L. (1959). *Manual for the Edwards Personal Preference Schedule.* New York, NY: Psychological Corporation.

[B35] Erpic S. C. (2013). The role of teachers in promoting students’ motivation for physical education and physical activity: a review of the recent literature from a self-determination perspective. *Int. J. Phys. Educ.* 50 2–13.

[B36] Eysenck S. B. G. Eysenck H. J. E. Barrett P. T. (1985). A revised version of the psychoticism scale. *Personal. Individ. Differ.* 6 21–29. 10.1016/0191-8869(85)90026-1

[B37] Fetiskin N. P. Kozlov V. V. Manuylov G. M. (2002). *Sotsialno-psikhologicheskaya diagnostika razvitiya lichnosti i malykh grupp[Sociopsychological Diagnostics of Personality and Small Group Development].* Moscow: Institut psikhoterapii, 426–433.

[B38] García-Moya I. Bunn F. Jiménez-Iglesias A. Paniagua C. Brooks F. M. (2018). The conceptualisation of school and teacher connectedness in adolescent research: a scoping review of literature. *Educ. Rev.* 71 423–444. 10.1080/00131911.2018.1424117

[B39] García-Villanueva J. Moreno-García D. Hernández-Ramírez C. I. Gamba-Mondragón L. A. (2017). Masculinity and feminity measurement in physical education teachers. *Rev. Int. Med. Cienc. Activ. Fis. Deport.* 17 541–557. 10.15366/rimcafd2017.67.010

[B40] Georgiev M. (2016). Pupils’ attitudes towards professional-personal qualities of the physical education teacher. *Res. Kinesiol.* 44 38–44.

[B41] Goldberg L. (1981). “Language and individual differences: the search for universals in personality lexicons,” in *Review of Personality and Social Psychology*, 2 Edn, ed. Wheeler L. (Beverly Hills, CA: Sage Publication), 141–165.

[B42] Göncz L. (2017). Teacher personality: a review of psychological research and guidelines for a more comprehensive theory in educational psychology. *Open Rev. Educ. Res.* 4 75–95. 10.1080/23265507.2017.1339572

[B43] Gough H. G. (1957). *Manual for the CPI, California Psychological Inventory.* Palo Alto: Calif Consulting Psychologists Press.

[B44] Gough H. G. Heilbrun A. B. J. (2012). *Adjective List. Manual – Edition from* 1983. Warsaw: Psychological Tests Laboratory of the Polish Psychological Society.

[B45] Hassan A. Shelvam P. V. Bhat J. A. Wani M. A. (2016). Big five personality factors among physical education and non-physical education teachers. *Int. Educ. Res. J.* 2 73–74.

[B46] Hattie J. (2009). *Visible Learning: a Synthesis of Over 800 Meta-Analyses Relating to Achievement.* London: Routledge.

[B47] Helmke A. (2017). *Unterrichtsqualität und Lehrerprofessionalität: Diagnose, Evaluation und Verbesserung des Unterrichts.* Seelze-Velber: Friedrich.

[B48] Holland J. L. (1994). *Self-Direct Search Form R: 1994 Edition.* Odessa, FL: Psychologocial Assessment Resources.

[B49] Holland J. L. (1997). *Making Vocational Choices: A Theory of Vocational Personalities and Work Environments.* Odessa, F.L: Psychological Assessment Research Inc.

[B50] Holtz P. Kronberger N. Wagner W. (2012). Analyzing internet forums: a practical guide. *J. Med. Psychol.* 24 55–66. 10.1027/1864-1105/a000062

[B51] Hosein Razavi S. M. Samimi A. J. Mohammadi S. H. Salehani F. T. (2012). Analysis of the relationship between entrepreneurial organizational culture & personality attributes of sport teachers: evidence from mazandaran province in Iran. *Middle East J. Sci. Res.* 11 201–208.

[B52] Jahangiri M. (2016). Teacher’ personality and students’ learning motivation. *Acad. J. Psychol. Stud.* 5 208–214.

[B53] John O. P. Naumann L. P. Soto C. J. (2008). “Paradigm shift to the integrative big five trait taxonomy: History, measurement, and conceptual issues,” in *Handbook of Personality: Theory and Research*, 3rd Edn, eds John O. P. Robins R. W. Pervin L. A. (New York, NY: The Guilford Press), 114–158.

[B54] Kell H. J. (2019). *Do Teachers’ Personality Traits Predict Their Performance? A Comprehensive Review of the Empirical Literature From 1990 to 2018.* Hoboken: Wiley.

[B55] Keller M. Neumann K. Fischer H. (2013). “Teacher Enthusiasm and Student Learning,” in *International Guide to Student Achievement*, ed. Hattie J. (New York, NY: Routledge), 247–250.

[B56] Khalilzadeh S. Khodi A. (2018). Teachers’ personality traits and students’ motivation: a structural equation modeling analysis. *Curr. Psychol.* 1–16. 10.1007/s12144-018-0064-8

[B57] Killian C. Kinder C. Woods A. (2019). Online and blended instruction in k–12 physical education: a scoping review. *Kinesiol. Rev.* 8 1–20. 10.1123/kr.2019-0003

[B58] Kim L. E. Dar-Nimrod I. MacCann C. (2018). Teacher personality and teacher effectiveness in secondary school: personality predicts teacher support and student self-efficacy but not academic achievement. *J. Educ. Psychol.* 110 309–323. 10.1037/edu0000217

[B59] Kim L. E. Jörg V. Klassen R. M. (2019). A meta-analysis of the effects of teacher personality on teacher effectiveness and burnout. *Educ. Psychol. Rev.* 31 163–195. 10.1007/s10648-018-9458-2 30930595 PMC6407857

[B60] Krause A. (2003). Lehrerbelastungsforschung - Erweiterung durch ein handlungspsychologisches Belastungskonzept. *Z. Pädagog.* 49 254–273.

[B61] Kunter M. Kleickmann T. Klusmann U. Richter D. (2013a). “The development of teachers’ professional competence,” in *Cognitive Activation in the Mathematics Classroom and Professional Competence of Teachers: Results from the COACTIV Project*, eds Kunter M. Baumert J. Blum W. Klusmann U. Krauss S. Neubrand M. (New York, NY: Springer), 63–77. 10.1007/978-1-4614-5149-5_4

[B62] Kunter M. Klusmann U. Baumert J. Richter D. Voss T. Hachfeld A. (2013b). Professional competence of teachers: effects on instructional quality and student development. *J. Educ. Psychol.* 105 805–820. 10.1037/a0032583

[B63] Lacala Z. Noworol C. Beauvale A. (2002). *Self-directed Search kit - version S,” in A textbook for career advisor. A Polish adaptation of S-1994 version of SDS.* Warszawa: Ministerstwo Pracy i Polityki Spolecznej.

[B64] Lara Cantú M. A. (1993). *Inventario de Masculinidad - Femineidad : IMAFE: Manual.* México, D.F: El Manual Moderno.

[B65] Lauritsalo K. Sääkslahti A. Rasku-Puttonen H. (2015). School PE through Internet discussion forums. *Phys. Educ. Sport Pedagogy* 20 17–30. 10.1080/17408989.2013.788144

[B66] Leary T. (2004). *Interpersonal Diagnosis of Personality. A Functional Theory and Methodology for Personality Evaluation.* Eugene: Wipf & Stock.

[B67] Makhmutova R. K. Baranov A. A. Ovechkin V. P. (2017). Physical education specialists’ mental burnout versus personality development and professional mastery rates. *Teor. Praktika Fiz. Kult.*

[B68] Mantu B. Montu B. (2014). A comparative study of personality traits between physical education and general education teachers of Assam. *Res. J. Phys. Educ. Sci.* 2 17–19.

[B69] Maryam S. F. Shahram A. Fakhrodin A. F. Mobarake B. Islamic A. (2017). Relationship between burnout with mental health and personality traits among physical education teachers. *Eur. J. Exp. Biol.* 2 2140–2144.

[B70] Maslach C. Jackson S. E. Leiter M. P. (1996). *Maslach Burnout Inventory Manual.* Palo Alto, CA: Consulting psychologists press.

[B71] Mayr J. (2011). “Der Persönlichkeitsansatz in der Lehrerforschung,” in *Handbuch der Forschung zum Lehrerberuf*, eds Terhart E. Bennewitz H. Rothland M. (Münster: Waxmann), 125–148.

[B72] Mays N. Roberts E. Popay J. (2001). “Synthesising research evidence,” in *Studying the Organisation and Delivery of Health Services: Research Methods*, eds Fulop N. Allen P. Clarke A. Black N. (London: Routledge), 188–220.

[B73] McAdams D. Pals J. L. (2006). A new big five: fundamental principles for an integrative science of personality. *Am. Psychol.* 61 204–217. 10.1037/0003-066X.61.3.204 16594837

[B74] McCrae R. R. Costa P. T. (2004). A contemplated revision of the NEO five-factor inventory. *Personal. Individ. Differ.* 36 587–596. 10.1016/S0191-8869(03)00118-1

[B75] McEvoy E. MacPhail A. Heikinaro-Johansson P. (2015). Physical education teacher educators: a 25-year scoping review of literature. *Teach. Teach. Educ.* 51 162–181. 10.1016/j.tate.2015.07.005

[B76] Messing M. (1979). Die persoenlichkeit des sportlehrers aus der sicht des schuelers. *Int. J. Phys. Educ.* 16 23–30.

[B77] Miethling W.-D. Gieß-Stüber P. (2007). *Beruf: Sportlehrer/in - Über Persönlichkeit, Kompetenzen und Professionelles Selbst von Sport- und Bewegungslehrern.* Baltmannsweiler: Schneider Hohengehren.

[B78] Munn Z. Peters M. D. J. Stern C. Tufanaru C. McArthur A. Aromataris E. (2018). Systematic review or scoping review? Guidance for authors when choosing between a systematic or scoping review approach. *BMC Med. Res. Methodol.* 18:143. 10.1186/s12874-018-0611-x 30453902 PMC6245623

[B79] Nie Y. Lau S. (2009). Complementary roles of care and behavioral control in classroom management: the self-determination theory perspective. *Contemp. Educ. Psychol.* 34 185–194. 10.1016/j.cedpsych.2009.03.001

[B80] Perera H. N. Granziera H. McIlveen P. (2018). Profiles of teacher personality and relations with teacher self-efficacy, work engagement, and job satisfaction. *Personal. Individ. Differ.* 120 171–178. 10.1016/j.paid.2017.08.034

[B81] Pervin L. A. Cervone D. (2008). *Personality. Theory and Research.* Hoboken, NJ: John Wiley & Sons.

[B82] Peters M. Godfrey C. McInerney P. Soares C. Khalil H. Parker D. (2015). “Methodology for JBI scoping reviews,” in *The Joanna Briggs Institute Reviewers’ Manual 2015*, ed. Aromataris E. (Adelaide: The Joanna Briggs Institute), 1–24.

[B83] Pourghaz A. Jenaabadi H. Ghaeninejad Z. (2016). Personality types and sense of humor and their association with teachers’ performance improvement. *New Educ. Rev.* 46 247–259. 10.15804/tner.2016.46.4.21

[B84] Rammstedt B. Danner D. Soto C. J. John O. P. (2018). Validation of the short and extra-short forms of the big five inventory-2 (BFI-2) and their german adaptations. *Eur. J. Psychol. Assess.* 1–13. 10.1027/1015-5759/a000481

[B85] Richards K. A. R. Washburn N. Carson R. L. Hemphill M. A. (2017). A 30-year scoping review of the physical education teacher satisfaction literature. *Quest* 69 494–514. 10.1080/00336297.2017.1296365

[B86] Robinson D. B. (2018). Religion as an other(ed) identity within physical education: a scoping review of relevant literature and suggestions for practice and inquiry. *Eur. Phys. Educ. Rev.* 25 491–511. 10.1177/1356336X17747860

[B87] Sallis J. F. McKenzie T. L. (1991). Physical education’s role in public health. *Res. Q. Exerc. Sport* 62 124–137. 10.1080/02701367.1991.10608701 1925034

[B88] Samdal O. Nutbeam D. Wold B. Kannas L. (1998). Achieving health and educational goals through schools – a study of the importance of the school climate and the students’ satisfaction with school. *Health Educ. Res.* 13 383–397. 10.1093/her/13.3.383

[B89] Schaarschmidt U. (2004). “Fit für den Lehrerberuf? Psychische Gesundheit von Lehramtsstudierenden und Referendaren,” in *Ein neues Bild vom Lehrerberuf? Pädagogische Professionalität nach Pisa – Beiträge zur Reform der Lehrerbildung*, eds Beckmann U. Brandt H. Wagner H. (Weinheim: Beltz), 100–115.

[B90] Schaarschmidt U. (2005). “Situationsanalyse,” in *Halbtagsjobber? Psychische Gesundheit im Lehrerberuf – Analyse eines veränderungsbedürftigen Zustands*, ed. Schaarschmidt U. (Weinheim: Beltz), 41–71.

[B91] Schaufeli W. Enzmann D. (1998). *The Burnout Companion To Study And Practice: A Critical Analysis.* Milton Park: Taylor & Francis.

[B92] Schaufeli W. Leiter M. Maslach C. (2009). Burnout: 35 Years of research and practice. *Career Dev. Int.* 14 204–220. 10.1108/13620430910966406

[B93] Senn V. Kornexl E. Greier K. (2017). Persönlichkeitsmerkmale und Kompetenzen von Sportlehrkräften und deren Einfluss auf die Motivation von Schülerinnen und Schülern. *Bewegung Sport* 1 18–23.

[B94] Sevinç L. (2005). *PERİ kişilik envanteri teknik kitapcığı (Technical Booklet of PERI Personality Inventory).* Istanbul: Assesment Systems Yayınları.

[B95] Sjöberg L. (2015). Correction for faking in self-report personality tests. *Scand. J. Psychol.* 56 582–591. 10.1111/sjop.12231 26043667

[B96] Sperka L. Enright E. (2017). The outsourcing of health and physical education: a scoping review. *Eur. Phys. Educ. Rev.* 24 349–371. 10.1177/1356336X17699430

[B97] Swann W. B. J. Bosson J. K. (2010). “Identity Negotiation,” in *Handbook of Personality: Theory and Research*, eds John O. P. Robins R. W. Pervin L. A. (New York, NY: Guilford).

[B98] Szczepanski S. (2012). The physical education teacher in self and peer assessment. *J. Phys. Educ. Health* 2 13–29.

[B99] Thompson S. W. (2010). *The Caring Teacher: A Multiple Case Study That Looks at What Teachers Do and Believe about Their Work With At-Risk Students.* Ph.D. Dissertation, University of Nebraska, Lincoln.

[B100] Voll S. (2006). “Welche Kompetenzen benötigt der Sportlehrer? Gegenwärtige Schülererwartungen an den Sportlehrer,” in *Der Sportlehrerberuf im Wandel: Jahrestagung der dvs-Sektion Sportsoziologie in Zusammenarbeit mit den Sektionen Sportpädagogik und Sportgeschichte vom 17.-19. November 2005 in Tübingen*, ed. Voll S. (Tübingen: Czwalina).

[B101] Wayne A. J. Youngs P. (2003). Teacher characteristics and student achievement gains: a review. *Rev. Educ. Res.* 73 89–122. 10.3102/00346543073001089

[B102] Weinert F. E. Helmke A. (1996). *Der gute Lehrer: Person, Funktion oder Fiktion? Die Institutionalisierung von Lehren und Lernen* (Weinheim: Beltz), 223–233.

[B103] Williams K. D. Govan C. L. Croker V. Tynan D. Cruickshank M. Lam A. (2002). Investigations into differences between social- and cyberostracism. *Group Dyn. Theor. Res. Pract.* 6 65–77. 10.1037/1089-2699.6.1.65

[B104] Zalech M. (2011a). Elimination of negative character features as an element of building a positive image of physical education teacher. *Sport Tour.* 18 206–209. 10.2478/v10197-011-0016-4

[B105] Zalech M. (2011b). Positive personality traits as an element of creating the image of a physical education teacher. *Balt. J. Health Phys. Act.* 3 121–128. 10.2478/v10131-011-0012-6

[B106] Zalech M. Rutkowska K. (2014). The image of a physical education teacher as seen by school community. *Lase J. Sports Sci.* 5 13–24. 10.1515/ljss-2016-0028 19574099

