# Supplementary material for: Inside Out: A Scoping Review on the Physical Education Teacher’s Personality
Source: Front Psychol. 2019 Nov 8;10:2510. doi: 10.3389/fpsyg.2019.02510 (PMC6856217; doi:10.3389/fpsyg.2019.02510)
Supplement: Supplementary file 1 [file Table_1.docx]

**Supplementary Table 1. Studies before 1991**

| **Author (Year)**  **Origin** | **Study Design/**  **Method**  **Sample** | **Aim** | **Personality Inventory** | **Personality Under-standing** | **Main Results** |
| --- | --- | --- | --- | --- | --- |
| Bahneman (1973)  USA | *cross-sect./ quant./qual.  *42 exp. PETs | To investigate the relations. betw. pers. char. & verbal behavior of exp. PETs (teacher-stud. verbal interaction) | quest.: California Psychological Inventory (*CPI*) (Gough, 1957) = 480 items | interpersonal behavior | *PETs using indirect verbal behavior = *sociable, tolerant, flexible, have a sense of well-being* / PETs using direct verbal behavior = *dominant, responsible, self-accepting, intellectually efficient, psychologically minded*  *PETs with high rates of interaction = *sociable & flexible*  *PETs with lower rates of interaction (silent classes) = *dominant & self-accepting* |
| Friedmann (1983)  Germany/ Israel | *cross-sect./quant.  *500 stud. – age 12-14 | To show the profile of the average/real & ideal PET from the stud.’ viewpoint & deduce ideas for changing attitudes in PET training | quest.: self-dev. (NN) = 20 items (PET char. traits, teaching method, behavior) + description of  ideal PET + 3 most imp. char. traits | not specifically determinable / own understanding | *profile of average/real PET: very good average marks in: *prof. competence, sportsmanship, pleasant appearance, self-confidence*  🡪 PET must *cause joy & pleasure, has to be an example in skills & behavior, should have fun with stud., should have authority & be admired*  *qualities of ideal PET: *prof. knowledge & competence* (53%); *understanding of stud.* (32%); *sportsmanship & ability to give interesting lesson*s (26%) |
| Gruber (1960)  USA | *cross-sect./quant.  *203 undergrad. m. stud. majoring in PE, 112 PETs (112 m.) | To determine if undergrad. m. stud. majoring in PE project the same pers. traits & attitudes towards teaching as grad. m. PETs (successful vs. unsuccessful) | quest.: Guilford Zimmermann Temperament Survey (*GZTS*) (Guilford and Zimmerman, 1949) = 300 items | trait psychological & temperament | *no diff. betw. mean scores (pers. & attitudes) of PETs & stud. or betw. successful & unsuccessful PETs & stud.  *diff. on item level  🡪 88 items discriminate betw. most successful PETs & stud.  🡪 77 discriminate betw. least successful PETs & stud. |
| Hale  (1973)  USA | *cross-sect./quant.  *122 sec. school & coll. football coaches, 32 handball players (= control group) | To determine relations. of personal background, educational exp., military background & coaching exp. of high school & coll. football coaches’ pers. char. & compare to control group | quest.: Adjective Check List (*ACL*) (Gough and Heilbrun, 1965) = 300 adjectives | trait  psychological | *football coaches scored higher on *nurturance* compared to handball players  * diff. betw. PE coaches with uni. degree & PE coaches without: qualified coaches scored higher on *dominance* & *aggression* & lower on *deference* *no diff. 1) betw. football coaches with intensive & those with less intensive liberal arts coursework; 2) betw. football coaches with previous military exp. & those with none; 3) betw. beginning & exp. coaches; 4) betw. head coaches & assistant coaches |
| Holmen & Parkhouse  (1981)  USA | *cross-sect./quant.  *49 suburban & inner-city high school PETs (25 m.) | To determine to what extent & how the individual pers. (self-concept; real vs. ideal self; functionality of pers. char.) is determined by the working environment | quest.: self-dev. (Holmen Adjective Check List - *HACL*) adapted from *ACL* (Gough and Heilbrun, 1965)) = 105 items | self, vocational & trait psychological | *individuals’ pers. linked to nature of environment  🡪 suburban PETs have more accurate self-concepts & are seen more like their ideal selves by their colleagues than inner-city PETs *individuals exp. in a given work setting are not necessarily seen by themselves or their colleagues as better adapted to that environment than those not working there  🡪 suburban PETs scored higher on suburban & inner-city functionality scales  🡪 adj. describing functional char. in suburban setting more socially-oriented in inner-city environment more survival-oriented |
| Kane  (1975)  UK | *cross-sect./quant.  *852 PETs (no info regarding gender or age distribution) | To collect PETs’ perceived views on the relative importance of certain pers. char. for effective teaching in PE & compare regarding gender & age | quest.: NN -  adapted from existing pers. inventory (Anderson, source missing) = 24 items | interpersonal; behavioral; situational;  trait  psychological | *PETs consider all char. except *teaching family background* as relatively imp.  🡪 top 3: *ability to 1) gain respect of stud., 2) communicate ideas, 3) inspire confidence*  🡪 lowest 3: *1) good academic record, 2) desire to improve the world & society, 3) teaching family background*  *no diff. betw. mean factor scores (personal education, social concern, rapport) for m. & f. but for age groups (younger PETs rated higher) |
| Lipkovich (1977)  USA | *cross-sect./quant.  *94 f. PETs, 124 f. OSTs (in total 81 elem., 87 sec. school, 50 uni.) | To determine if there is an existing pattern of simil. among pers. var. *achievement, aggression, dominance* of f. PETs & OSTs at diff. school levels (elem., sec., coll.) & compare with norm | quest.: Edwards Personal Preference Scale (*EPPS*) (Edwards, 1959) *=* 210 items | human needs system theory | *no diff. betw. f. elem., sec. & coll. teachers (regardless of subject) for *achievement* & *aggression* but for *dominance /* coll. teachers diff. from elem. & sec. teachers but no diff. betw. elem. & sec. teachers  *no diff. betw. PETs & OSTs (regardless of school level) for *achievement, aggression, dominance*  *no diff. betw. f. elem., sec., coll. PETs & OSTs for *achievement* & *dominance* but for *dominance* betw. coll. PETs & OSTs & betw. coll. & sec. OSTs  *scores of f. elem., sec., coll. PETs & OSTs average compared to norm for *achievement* & *aggression* but for *dominance* coll. PETs & OSTs score higher |
| Messing (1979)  Germany | *cross-sect./quant.  *1582 stud. – 14-16 years | To examine pers. profiles of PETs from stud. view & identify char. pos. or neg. influencing stud. interests | quest.: self-dev. (NN) *=* 29 items PET char. + 29 items PET behavior | own understanding/not specifically determinable | *desired PET*: integrated, student-oriented teaching (sporty/professional, companionable, provides assistance/safety)* vs. undesired PET: *cannot take criticism, unamenable to personal problems, not interested in stud.’ opinions, puts pressure on stud., dominant* *relations. betw. stud.’ image of PET’s pers. & stud.’ desired teaching organization/style 🡪 stud. whose PET’s pers. resembles their desires satisfied with teaching organization |
| Phillips (1985)  USA | *cross-sect./quant.  *18 PETs, 144 stud. – 5^th^-8^th^ grade | To analyze the relations. of PETs’ pers. & a) PETs’ process behavior in PE, b) stud. behavior & c) stud. achievement gains | quest.: Cattell Person. Factor (*16 PF*) Quest. (Cattell et al., 1970) = 187 items (only for PETs) | trait psychological | *pers. traits *assertiveness, expediency, questioning, imaginativeness, genuineness, confidence, experimenting* relate with PET & stud. behavior in PE (PETs high on these exhibit more knowledge, utilize objectives & testing more effectively, instruct more flexible & appropriate, provide more planned instruction & feedback) *most consistent, meaningful relations. with teacher & stud. behavior = *expediency, questioning, imaginativeness, experimenting, independence* *PETs high in *assertiveness, questioning, imaginativeness* provide stud. with more time on task & higher quality of practice time  *stud. of PETs high in *independence*, *assertiveness*, *questioning*, *imaginativeness* learn more |
| Rider (1973)  USA | *cross-sect./quant.  *40 PE majors (senior & sophomore), 40 coll. & 40 sec. school PETs | To compare the pers. traits of PE senior majors, PE sophomore majors at uni. with coll. or sec. school PETs & compare the four PE groups with norm group & consider gender diff. | quest.: Cattell Person. Factor (*16PF*) Quest. (Cattell et al., 1970) = 187 items | trait psychological | *7/16 pers. traits diff. betw. the 4 groups (5 only consid. m., 2 only consid. f., 1 consid. m. & f.)  🡪 comparison of all f. groups = more simil. than diff. in the pers. traits  🡪 comparison of m. & f. in the individual groups revealed simil. patterns of pers. traits  🡪 groups differed from established norms in several pers. traits (m. sophomore majors most simil. & m. PE majors least simil. to the norm)  🡪 comparison of groups: coll. PE majors & coll. PETs highest degree of simil. / coll. PE majors & sec. PETs lowest degree of simil.  🡪 senior PE majors more *happy-go-lucky* & *assertive* than coll. PETs; sec. school PETs more *conscious* than coll. PETs, senior PE majors & sophomore PE majors; m. PETs more *tough-minded* than f. PETs |
| Srokosz (1988)  Poland | *cross-sect./quant.  *54 PETs (27. m.) – vocational schools | To examine prof. activities of PETs in regard to proceedings within a lesson & their relations. with pers. + compare to Czech coaches | quest.: Eysenck Pers. Quest. (*EPQ-R S*) (Eysenck et al., 1985) = 48 Items  & Cattell *16 PF* (Cattell et al., 1970) = 187 items | trait psychological | *PETs mainly *extraverted* (N = 14; 6 f., 8 m.) or *balanced* (N = 12; 8 m., 4 f.), only 4 *neurotic*  *compared to Czech coaches: PETs *less independent in thinking & progressive, more trustful, open, sociable, neurotic, revealing weaker ego, lacking perseverance, dependent, changeable in feelings, following group principles & conventions more* 🡪 surpass coaches in inclination to *dominance & aggression* *sex diff. more decisive for differentiating PET's lesson proceedings than pers. type  *PETs’ verbal activities more important for lesson proceedings than PETs’ motor activities & relations. betw. verbal activitites & PETs’ pers. types  *distribution of didactic interactions less clearly related to PETs’ pers. types |
| Svoboda (1982)  Czech Republic | *cross-sect./quant./qual.  *21 grammar & basic school PETs (12 m.) | To examine the pers. & activity of PETs in PE lessons | quest.: EPI (Eysenck, 1963), I-C-L (Leary, 1976), SPIDO (Miksik, 1980) + interview + observation | temperament, trait psychological and interpersonal | *no mean PET but individual personalities capable of attaining educational aims by means of diff. capacities  🡪 most represented category = *sanguine temperament* (N = 9); in general tendency to *extraversion* & *stability*  🡪 SPIDO: tendency to a *self-reliant* & *tenacious* pers.  🡪 I-C-L: quite unusual distribution; atypical for PETs & diff. compared to norm; 2 tendencies of PETs = *protective* & *competitive* **affiliation, conformity, modesty, responsibility =* very high*; dominance, criticism* = very low *total vs. ideal type = *dependent* vs. *protective* pers.  *PET’s activity level in class relatively high |

| Svoboda (1990)  Czech Republic | *cross-sect./quant.  *249 PETs (41 Czech, 60 Bulgarian, 54 Polish, 94 Hungarian) | To describe PETs’ pers. traits, temperament var. of PETs & show relations. betw. PET’s pers. & behavior & to compare countries | quest.: EPI (Eysenck, 1963), Leary (Leary, 1976), SPIDO (Miksik, 1980) | temperament, interpersonal & trait psychological | *majority belonging to 2 interpersonal pers. types: *autocratic* or *assertive* pers. (= indicated as ideal type) *all types of temperament can be found among PETs; majority of *stable* teachers, especially *sanguine* types of temperament (those without distinct temperament = *stable, extrovert*) *no diff. betw. various types of temperament & PETs’ structure of interactional behavior  *active social learning & role play irrespective of temperament types of pers.  *PETs are not able to use personal communication situations with stud. successfully & require help |
| --- | --- | --- | --- | --- | --- |
| Tancing  (1988)  Not known | *cross-sect./quant.  *651 stud. – 13-14 years (7^th^-8^th^ grade) | To determine profiles of average PETs & coaches from stud. viewpoint, compare them & deduce suggestions for changing & sophisticating the teaching process | quest.: self-dev. (NN) adapted form of Friedman’s Quest. (see (Friedmann, 1983)) *=* 20 personal traits | town understanding/not specifically determinable | *average marks for PETs in all items lower than coaches  *best marks very similar betw. groups: *sportsmanship, good discipline, good working habits, good human relations, prof. competence*  *diff.: coaches best mark *ability to arise enthusiasm for sport* only 8^th^ rank for PETs  *marks on *interesting lessons* lower for PETs  *both relatively low marks on *ability to stimulate independent. thinking, openness to stud.’ suggestions* & *to criticism* |
| Thorpe  (1958)  USA | *cross-sect./quant.  *100 PETs, 100 undergrad./senior majors, 55 grad. uni. stud. (all f.) | To determine if there is an existing pattern of simil. of pers. var. among diff. part. in PE group (successful f. PETs, undergrad., grad. stud.) & compare PE group with norm group | quest.: Edwards Personal Preference Scale (*EPPS*) (Edwards, 1954) *=* 210 items | human needs system theory  (Murray/  Edwards) | *diff. betw. PE group & norm group in 9/15 var.  🡪 PE group higher in: *deference, order, dominance, endurance*  🡪 PE group lower in: *autonomy, succorance, nurturance, heterosexuality, aggression*  *pattern of simil. of pers. var. among part. in PE group  *group diff. within PE group smaller than diff. betw. PE group & norm |

**Legend of abbreviations**

| **Study Design/Method**  **Sample** | **Aim** | **Personality Inventory** | **Main Results** |
| --- | --- | --- | --- |
| coll. = collegiate  cross-sect.= cross-sectional  elem. = elementary  exp. = experience(d)  f. = female  grad. = graduate  m. = male  OST = Other Subject Teacher  PE = Physical Education  PET = Physical Education Teacher  prim. = primary  quant. = quantitative  qual. = qualitative  sec. = secondary  stud. = students  undergrad. = undergraduate  uni. = university | betw. = between  char. = characteristics  coll. = collegiate  diff. = difference(s)  elem. = elementary  exp. = experience(d)  f. = female  grad. = graduate  m. = male  neg. = negative(ly)  part. = participants  pers. = personality  PE = physical education  PET = Physical Education Teacher  pos. = positive(ly)  prof. = professional  relations. = relationship(s)  sec. = secondary  simil. = similarit(y)ies  stud. = student(s)  undergrad. = undergraduate  var. = variables  vs. = versus | char. = characteristics  imp. = importan(t)ce  pers. = personality  PET = Physical Education Teacher  quest. = questionnaire  self-dev. = self-developed | adj. = adjectives  betw. = between  char. = characteristics  coll. = collegiate  consid. = considering  corr. = correlation(s)  diff. = difference(s)  elem. = elementary  exp. = experience(d)  f. = female  imp. = importan(t)ce  m. = male  OST = Other Subject Teacher  part. = participants  PE = physical education  pers. = personality  PET = Physical Education Teacher  pos. = positive(ly)  prof. = professional  relations. = relationship(s)  sec. = secondary  simil. = similar(ities)  stud. = student(s)  undergrad. = undergraduate  uni. = university  var. = variables  vs. = versus |

**References**

Bahneman, C.P. (1973). The relationship between personality and verbal behavior of physical education teachers. *Physical Educator* 30**,** 144-145.

Cattell, R.B., Eber, H.W., and Tatsuoka, M.M. (1970). *Handbook for the sixteen personality factor questionnaire (16 PF).* Champaign, Ill.: Institute for Personality and Ability Testing.

Edwards, A.L. (1954). *Manual for the Edwards Personal Preference Schedule.* New York: Psychological Corporation.

Edwards, A.L. (1959). *Manual for the Edwards Personal Preference Schedule.* New York: Psychological Corporation.

Eysenck, H.L. (1963). *Manual of the Eysenck Personality Inventory.* London: University of London Press.

Eysenck, S.B.G., Eysenck, H.J.E., and Barrett, P.T. (1985). A revised version of the psychoticism scale. *Personality and Individual Differences* 6**,** 21-29. doi: 10.1016/0191-8869(85)90026-1.

Friedmann, E.D. (1983). The pupil's image of the physical education teacher and suggestions for changing attitudes in teacher training. *International journal of physical education* 20(2)**,** 15-18.

Gough, H.G. (1957). *Manual for the CPI, California psychological inventory.* Palo Alto: Calif Consulting Psychologists Press.

Gough, H.G., and Heilbrun, A.B. (1965). *The adjective check list manual.* Palo Alto, CA: Consulting Psychologists Press.

Gruber, J.J. (1960). Personality traits and teaching attitudes. *Research Quarterly of the American Association for Health, Physical Education and Recreation* 31(3)**,** 434-439.

Guilford, J.P., and Zimmerman, W.S. (1949). *The Guilford-Zimmerman Temperament Survey. [Test booklet and manual].* Oxford, England: Sheridan Supply Co.

Hale, B.D. (1973). *The Relationships of Selected Personality Characteristics to the Background Experiences of Football Coaches.* Master Master, Pennsylvania State University.

Holmen, M.G., and Parkhouse, B.L. (1981). Relationship between working environment, self-concept, real-ideal self discrepancy, and functionality in physical education teachers. *Research Quarterly for Exercise and Sport* 52(3)**,** 311-323.

Kane, J.E. (1975). "Perception of personal characteristics by PE teachers and coaches," in *Readings in Sports Psychology 2,* ed. H.T.A. Whiting. (London: Lepus).

Leary, T. (1976). *Dotazník interpersonální diagnózy - ICL : příručka pro administraci, interpretaci a vyhodnocování testu.* Bratislava: Psychodiagnostické a didaktické testy.

Lipkovich, M.K. (1977). *A Comparison of Personality Variables of Achievement, Aggression and Dominance Among Female Physical Education Teachers at the Elementary, Secondary and Collegiate Levels.* PhD Dissertation, West Virginia Univeristy.

Messing, M. (1979). Die Persoenlichkeit des Sportlehrers aus der Sicht des Schuelers. *International journal of physical education* 16(3)**,** 23-30.

Miksik, O. (1980). Zjistovani interakcnich struktur osobnosti. *Bulletin Vyzkumncho ustavu penologickeho* 1.

Phillips, D.A., Carlisle, C.S., Hautala, R., and Larson, R. (1985). Personality traits and teacher–student behaviors in physical education. *Journal of Educational Psychology* 77(4)**,** 408-416.

Rider, V.J.E. (1973). *Personality Traits of Physical Education Teachers and Majors.* PhD Dissertation, University of Utah.

Srokosz, W. (1988). "Proceedings within a lesson in reference to the physical education teacher's personality," in *Coaches and physical education teachers training in socialist countries: a collection of studies in the international scientific cooperation (task no. 6),* ed. B. Svoboda. (Prague: Central Committee of the Czechoslovak Union of Physical Education), 189-166.

Svoboda, B. (Year). "Interactional personality profiles of physical education teachers", in: *International Symposium on Research in School Physical Education*, ed. R. Telama: Foundation for Promotion of Physical Culture and Health), 65-74.

Svoboda, B. (1990). "Teacher training in view of investigations on teacher personality and activities," in *Physical education and life-long physical activity: the proceedings of the Jyvaskyla Sport Congress, June 17-22, 1989, at the University of Jyvaskyla, Finland* ed. R. Telama. (Jyvaskyla, Finland: Foundation for Promotion of Physical Culture and Health), 376-382.

Tancing, S. (1988). "Evaluating personal traits and teaching behaviour of physical education teacher and coach from the pupil's point of view," in *The physical education teacher and coach today. Sportlehrer und Trainer heute. Bericht über den: AIESEP-Weltkongreß 22. - 26. August 1986 anläßlich der 600-Jahrfeier der Universität Heidelberg* eds. H. Rieder & U. Hanke. (Köln: Sport und Buch Strauß), 119-122.

Thorpe, J.A. (1958). Study of personality variables among successful women students and teachers of physical education. *Research Quarterly of the American Association for Health, Physical Education and Recreation* 29**,** 85-92. doi: 10.1080/10671188.1958.10612966.
